# Comparative Effects of Putrescine, Salicylic Acid, Citric Acid, and Methyl Jasmonate on Postharvest Quality and Storage Life of *Pleurotus ostreatus*

**DOI:** 10.1002/fsn3.70233

**Published:** 2025-04-30

**Authors:** Bilgehan Şahin, Beyhan Kibar, Hakan Kibar

**Affiliations:** ^1^ Occupational Health and Safety Coordination Office Bolu Abant Izzet Baysal University Bolu Türkiye; ^2^ Faculty of Agriculture, Department of Horticulture Bolu Abant Izzet Baysal University Bolu Türkiye; ^3^ Faculty of Agriculture, Department of Seed Science and Technology Bolu Abant Izzet Baysal University Bolu Türkiye

**Keywords:** color properties, natural compounds, nutritional properties, *Pleurotus ostreatus*, storage

## Abstract

This study was carried out to determine the effects of exogenous putrescine, salicylic acid, citric acid, and methyl jasmonate treatments at different doses (0.5 mM, 1.0 mM, and 2.0 mM) after harvest on quality properties, storage period, and nutrient content in *Pleurotus ostreatus* mushroom species, which ranks second in terms of production in the world and deteriorates rapidly after harvest and loses its quality. A total of 13 different treatments were examined in the study, and the necessary measurements and analyses (on the 7th and 14th days) were performed on the mushrooms stored at 4°C for 14 days. As a result of the research, it was determined that the postharvest quality characteristics changed significantly depending on treatments and storage periods. The results showed that postharvest treatments significantly influenced weight loss, dry matter, protein content, color, pH, and mineral composition during storage. Compared to the control, 0.5 mM methyl jasmonate was the most effective in preserving protein content (increased by 13.56%) and calcium content (increased by approximately threefold), while 2.0 mM citric acid reduced weight loss by 7.76% and increased zinc content by 57%. Principal component analysis and cluster analysis further confirmed that citric acid and methyl jasmonate treatments were superior in maintaining postharvest quality. It was concluded that 0.5 mM methyl jasmonate, 0.5 mM, 1.0 mM, and 2.0 mM citric acid treatments can be recommended as an alternative application method to reduce postharvest quality losses of *P. ostreatus*.

## Graphical abstract

This study investigated the effects of exogenous putrescine, salicylic acid, citric acid, and methyl jasmonate on the postharvest quality and storage life of *Pleurotus ostreatus* mushrooms. The findings revealed that citric acid and methyl jasmonate treatments were the most effective in reducing weight loss, maintaining protein and dry matter content, and preserving essential minerals such as potassium, phosphorus, and zinc. Overall, the results suggest that 0.5 mM methyl jasmonate and 0.5 mM–2.0 mM citric acid can be recommended as alternative treatments to extend the shelf life and enhance the postharvest quality of *P. ostreatus* mushrooms.

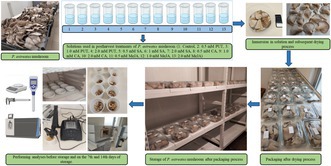

## 1 Introduction

Nowadays, with the better understanding of the value of mushrooms in terms of human nutrition and health, the production and consumption of cultivated mushrooms have increased rapidly all over the world and have become an important sector (Kalač 2013). Mushrooms are very rich in protein, minerals, vitamins, non‐starchy carbohydrates, and dietary fibers and have low cholesterol, calorie, and fat content (Kalač 2009). Mushroom proteins are of high quality and contain all the essential amino acids needed by humans (Kakon et al. 2012). In addition, mushrooms have very important functions for health due to the bioactive compounds they contain (Zhu et al. 2015).

Among the mushrooms cultivated today, *Pleurotus* species have a large production volume in world mushroom production. *Pleurotus* species rank second after *Agaricus bisporus* among the most produced mushroom species in the world (Royse 2014). *Pleurotus* mushrooms are often called “oyster mushrooms” around the world because they are oyster‐shaped. Among the *Pleurotus* species, *P. ostreatus* was the first to be cultured and the most commercially cultivated species in the world. *P. ostreatus* has been preferred worldwide for many years due to its high nutritional value and medicinal properties, as well as its characteristic taste, pleasant aroma, high productivity, resistance to diseases and pests, short life cycle, and its ability to be easily grown on a wide variety of agricultural–industrial wastes (Kibar et al. 2016). *P. ostreatus* contains many bioactive compounds with antioxidant properties such as polysaccharides, polyphenols, carotenoids, and tocopherols (Sarma et al. 2018). Also, it naturally contains lovastatin (mevinolin), which is used as the active ingredient of cholesterol‐lowering drugs (Prasad and Roy 2023).

However, edible mushrooms deteriorate very quickly after harvest and lose their quality (Liufang et al. 2024). The extremely rapid deterioration of fresh mushrooms is due to their high moisture content (85%–95%), which supports microbial activities, a high respiration rate, delicate texture, and high enzymatic activity (Zhang et al. 2018). Mushrooms have a very short shelf life of 3–4 days at room temperature. The rapid deterioration of mushrooms after harvest and their short shelf life cause difficulties in their distribution and marketing as fresh products and limit their consumption amount (Liufang et al. 2024). This situation is one of the biggest limitations in the commercialization of mushrooms. Therefore, extending the shelf life of mushrooms is a continuous quest for mushroom production and the supply chain (Akbarirad et al. 2013).

Physical parameters such as color, taste, texture, and odor, as well as general appearance, are the quality factors that most affect consumers' purchasing behavior regarding fresh mushrooms (Nasiri et al. 2018). Various morphological and physiological changes that occur after harvest make mushrooms unacceptable for fresh consumption. During the postharvest storage of mushrooms, quality losses such as weight loss (WL), stem elongation, cap opening, rotting, loss of flavor, decrease in nutritional content, formation of undesirable odors, color changes (darkening, browning, etc.), texture changes (softening, hardening, etc.), and microbial infections occur (Zhang et al. 2018). This often causes major economic losses and significantly reduces the commercial value of the mushroom (Song et al. 2019). For this reason, studies that can increase the storage period of mushrooms even by a few days are of great importance.

In recent years, natural compounds such as polyamines, salicylic acid (SA), citric acid (CA), and jasmonic acid have also been used in addition to different preservation techniques to reduce postharvest quality losses in mushrooms and extend their storage life. Studies on postharvest applications of these compounds and their effects on the storage duration and quality of various mushroom species have become increasingly prevalent. However, the effectiveness of these compounds varies depending on many factors such as species, variety, environmental conditions, application method, duration, and concentration. Besides, more studies on this subject are needed to transfer these applications to agricultural practice.

Polyamines, one of the substances used to protect or improve postharvest quality in products, are low‐molecular weight organic compounds found in almost all living organisms. Among the polyamines, putrescine (PUT) is generally found in the highest proportion (González‐Hernández et al. 2022). It has been reported that external polyamine applications in postharvest storage of fruits and vegetables delay ripening and aging, slow down fruit flesh softening, inhibit ethylene production and activity, control respiration rate, and affect quality characteristics such as fruit flesh firmness, WL, color, total soluble solids (TSS), and titratable acidity (Khan et al. 2007; Jhalegar et al. 2012; Palma et al. 2014).

SA is an endogenous plant growth regulator that functions in the regulation of physiological events in plants (Hayat et al. 2010; Jakhar et al. 2023). It constitutes a group of plant phenolics (Rivas‐San Vicente and Plasencia 2011). SA inhibits ethylene biosynthesis, delays aging, increases resistance to pathogens, and reduces decay (Yang et al. 2011). SA is a natural and safe compound with high potential to extend the postharvest storage life of fruits and vegetables and maintain their nutritional quality (Asghari and Aghdam 2010). SA applied externally to horticultural crops has been found to be effective in preserving postharvest quality (Asghari and Aghdam 2010; Dokhanieh and Aghdam 2016; Sen et al. 2024).

CA is an organic acid and increases the postharvest storage life of horticultural products. It is widely used as a preservative in the food industry. CA prevents the product from darkening by increasing the acidity of the medium (Garcia and Barrett 2002). The polyphenol oxidase (PPO) enzyme, which is associated with enzymatic darkening, was blocked by CA. It has been reported that postharvest CA application preserves the quality and extends the storage life of different horticultural crops (Gupta and Bhat 2016; Alali et al. 2023; Kibar et al. 2023, 2024).

Methyl jasmonate (MeJA) is a growth regulator that has attracted attention in recent years because it slows down postharvest ripening. MeJA is the methyl ester of jasmonic acid and is a fragrant compound found naturally in many plants. MeJA applications are reported to reduce postharvest respiration and ethylene synthesis (Zapata et al. 2014). It has been determined that MeJA applications protect postharvest quality in different horticultural crops (Meng et al. 2012; Fan et al. 2016a, 2016b; Faizy et al. 2021; Öztürk and Yücedağ 2021; Deshi et al. 2022).

Despite the growing interest in the use of these natural compounds, there is limited information on their comparative effects on *P. ostreatus* mushrooms during cold storage. While some studies have reported the effectiveness of PUT in reducing WL and delaying senescence in fresh produce (Jia et al. 2018; Motamedi et al. 2020), others have highlighted the role of SA in inhibiting ethylene production and maintaining postharvest quality in mushrooms (Dokhanieh and Aghdam 2016; Kibar et al. 2023). CA has been shown to prevent enzymatic browning and improve antioxidant activity in postharvest produce (Gupta and Bhat 2016; Alali et al. 2023), whereas MeJA has been linked to delayed ripening and enhanced stress tolerance in stored horticultural products (Meng et al. 2012; Fan et al. 2016a, 2016b).

This study aimed to investigate the effectiveness of exogenous applications of PUT, SA, CA, and MeJA in preserving the postharvest quality of *P. ostreatus* during cold storage. Given the rapid deterioration and short shelf life of this commercially valuable mushroom species, this research sought to address the key questions: (1) How do different postharvest treatments (PUT, SA, CA, and MeJA) affect the quality parameters (e.g., WL, dry matter, protein content, pH, color, and mineral composition) of *P. ostreatus* during storage at 4°C? (2) Which of the tested compounds and concentrations (0.5 mM, 1.0 mM, and 2.0 mM) is most effective in reducing postharvest quality losses and extending storage life? (3) Do these treatments mitigate specific postharvest physiological and biochemical changes, such as WL, enzymatic browning, and nutrient depletion? It was hypothesized that exogenous applications of PUT, SA, CA, and MeJA would significantly enhance the postharvest storage quality of *P. ostreatus* by reducing WL, maintaining dry matter and protein content, preventing color degradation, and preserving essential minerals.

## 2 Materials and Method

### 2.1 Material

Fresh mushroom samples of *Pleurotus ostreatus* (Jacq. ex. Fr.) Kummer used in the study were supplied by a commercial mushroom producer in Bolu, Türkiye. PUT, SA, and MeJA used in the study were purchased from Sigma‐Aldrich company, and CA was purchased from Akbel Kimya company.

### 2.2 Treatments and Experimental Design

In the study, a total of 13 different treatments using three different doses (0.5 mM, 1.0 mM, and 2.0 mM) of PUT, SA, CA, and MeJA were investigated (Table 1). PUT, SA, CA, and MeJA were not added to the control application. The doses of PUT, SA, CA, and MeJA used in this study were selected based on previous research investigating their effectiveness in preserving postharvest quality in mushrooms and other horticultural products. The concentrations of 0.5 mM, 1.0 mM, and 2.0 mM align with doses that have been widely applied in similar studies to delay senescence, reduce oxidative stress, maintain nutritional quality, and extend storage life (Asghari and Aghdam 2010; Meng et al. 2012; Dokhanieh and Aghdam 2016; Gupta and Bhat 2016; Fan et al. 2016a; Jia et al. 2018; Motamedi et al. 2020; Alali et al. 2023).

**TABLE 1:** Treatments discussed in the study, their contents, and abbreviations.

Treatment	Content	Abbreviation
1	Control	Control
2	0.5 mM Putrescine	0.5 mM PUT
3	1.0 mM Putrescine	1.0 mM PUT
4	2.0 mM Putrescine	2.0 mM PUT
5	0.5 mM Salicylic Acid	0.5 mM SA
6	1.0 mM Salicylic Acid	1.0 mM SA
7	2.0 mM Salicylic Acid	2.0 mM SA
8	0.5 mM Citric Acid	0.5 mM CA
9	1.0 mM Citric Acid	1.0 mM CA
10	2.0 mM Citric Acid	2.0 mM CA
11	0.5 mM Methyl Jasmonate	0.5 mM MeJA
12	1.0 mM Methyl Jasmonate	1.0 mM MeJA
13	2.0 mM Methyl Jasmonate	2.0 mM MeJA

The experiment was set up according to the completely randomized design (CRD) with three replications, and each replication included three packages containing 400 g of mushroom sample.

### 2.3 Sample Preparation, Postharvest Treatments, and Storage

The mushrooms were harvested at the commercial maturity stage, which was determined based on the typical morphological characteristics of *P. ostreatus*. Specifically, the mushrooms were collected when their caps were fully expanded but had not yet begun to curl upwards, ensuring optimal quality and freshness for postharvest analysis. Only mushrooms with intact, firm structures, uniform size, and no visible signs of decay or over‐maturity were selected for the study. After harvesting, *P. ostreatus* samples were immediately transferred to the laboratory within 2 h to minimize postharvest deterioration. The mushrooms were placed in ventilated plastic crates, which helped prevent excessive moisture accumulation and mechanical damage during transportation. The transfer was conducted under ambient conditions, with an approximate temperature of 15°C–20°C and relative humidity of 50%–60%. Upon arrival at the laboratory, the mushrooms were promptly sorted for uniformity and subjected to postharvest treatments. Morphological properties of the initial fresh mushroom sample (cap diameter, stem length, stem diameter, and average mushroom weight), dry matter, pH, ash, color values (L*, a*, b*, C*, and h°), TSS, total nitrogen, protein, and mineral contents (K, Mg, Ca, P, Fe, Mn, Cu, and Zn) were determined. Fresh mushroom samples were divided into 13 equal groups for applications. Then, the mushrooms were subjected to the treatments included in the study.

PUT, SA, CA, and MeJA solutions (0.5 mM, 1.0 mM, and 2.0 mM) were prepared at the doses discussed in the study. The treatment solutions were prepared by dissolving the respective chemicals in distilled water. For PUT, SA, and CA, the required concentrations (0.5 mM, 1.0 mM, and 2.0 mM) were prepared by directly dissolving the compounds in distilled water with continuous stirring until complete dissolution. MeJA, due to its low solubility in water, was first dissolved in 0.01% (v/v) Tween 20 as an emulsifier to ensure proper dispersion and then diluted with distilled water to obtain the desired concentrations. Postharvest treatments were performed by immersing mushroom samples in solutions. Mushroom samples were immersed in PUT, SA, CA, and MeJA solutions (2 L, 4°C) containing a 0.01% dose of Tween 20 used as an adhesive and left for 5 min. Control group samples were immersed in 2 L of distilled water at 4°C containing a 0.01% dose of Tween 20 used as an adhesive and kept for 5 min. After the immersion process, all samples were kept on drying papers for 30 min at room conditions (20°C–23°C and 50%–60% relative humidity) to remove excess water. 10 holes with a diameter of 1.0 mm were drilled on the lids of the transparent plastic containers (60 × 40 × 23 cm) to be used in mushroom preservation. Then, the mushroom samples were weighed to approximately 400 g and placed in transparent plastic containers and the lids were closed.

Samples packaged in three replicates were stored in a cold storage room at 4°C ± 0.5°C and 85% ± 5% relative humidity for 14 days. WL, dry matter, pH, ash, color, TSS, total nitrogen, protein, and mineral contents were determined in mushroom samples at 7‐day intervals (on the 7th and 14th days) during the storage period. No precooling was applied to *P. ostreatus* mushrooms before the postharvest treatments. This approach was chosen to simulate commercial postharvest handling practices and to evaluate the direct effects of the applied treatments on storage quality.

### 2.4 Morphological Characteristics, WL, Dry Matter, pH, TSS, Ash, and Color

The morphological characteristics of mushrooms (cap diameter, stem length, and stem diameter) were determined with a digital caliper. The average mushroom weight was detected using a precision balance with a sensitivity of 0.01 g.

Weight losses during storage were determined by weighing the mushrooms at the beginning of storage and at each analysis period using a precision balance with a sensitivity of 0.01 g. WL was calculated as % compared to the beginning using the following formula (Kim et al. 2006):

(1) $$\mathrm{WL}=\frac{\left(W_{i}-W_{f}\right)}{W_{i}}\times 100$$

where *W*_*i*_ = Weight of mushroom samples before storage, g; *W*_*f*_ = Weight of mushroom samples on the 7th and 14th days of storage, g.

Dry matter and ash contents of mushroom samples were determined according to the procedures of AOAC (1990). pH values of mushroom samples were measured using a digital pH meter (Thermo Scientific, Orion Star A111). TSS was measured with a hand‐held refractometer (ATC‐1, Atago, Japan). The color properties of the mushroom samples were determined by reading the L*, a*, b*, C*, and h° color values with a colorimeter (3NH NR60Cp, China).

### 2.5 Total Nitrogen, Protein, and Mineral Contents

The total nitrogen (N) content was determined according to the Kjeldahl method, and the protein content of mushrooms (Nx6.25) was calculated as described by AOAC (1990).

To determine the K, P, Mg, Ca, Fe, Mn, Cu, and Zn contents, mushroom samples were dried in the oven at 65°C until they reached constant weight. Afterward, dried mushroom samples were ground into powder by using a grinder (MC23200, Siemens, Germany). Subsequently, the samples were prepared for analysis according to the microwave digestion method. Mineral contents were determined using an Inductively Coupled Plasma‐Optical Emission Spectroscopy (ICP‐OES, Perkin‐Elmer Optima 7000 DV) device.

### 2.6 Statistical Analysis

The experiment was performed according to the CRD with three replications. The results were given as mean ± standard deviation. The data obtained were subjected to variance analysis using the JMP 16.0 statistical program. Postharvest treatment × storage period interaction and statistically significant differences among the means of postharvest treatments were determined by Tukey's HSD (Tukey's Honestly Significant Difference Test) multiple comparison test. An independent t‐test was performed to determine the differences between the means of the storage periods (7th and 14th days). Hierarchical cluster analysis (HCA) and principal component analysis (PCA) were performed to evaluate the similarities and differences among treatments based on postharvest quality parameters of *P. ostreatus* mushrooms. HCA was conducted using Ward's method with squared Euclidean distance as a measure of dissimilarity to group the treatments according to their effects on WL, protein content, TSS, pH, ash, dry matter, color attributes, and mineral composition. The results were visualized using a dendrogram to illustrate the clustering pattern among treatments. PCA was applied to reduce the dimensionality of the dataset and identify principal components (PCs) explaining the most variance in the data. Eigenvalues > 1 were considered significant in determining the number of PCs. The relationships among treatments and measured parameters were visualized using biplots, which facilitated the interpretation of key variables contributing to postharvest quality differentiation. All chemical analyses were conducted in triplicate. Measurements related to morphological characteristics were made in 10 repetitions.

## 3 Results and Discussion

### 3.1 Initial Properties of Fresh *P. Ostreatus* Mushroom

Some morphological and nutritional properties of fresh *P. ostreatus* mushroom before storage are presented in Table 2. Dry matter, protein, pH, TSS, and ash contents of the fresh mushroom sample were determined as 11.14%, 27.25%, 5.36, 3.20%, and 6.88%, respectively. Similar results regarding the morphological and nutritional properties of *P. ostreatus* were also reported by Tolera and Abera (2017), Turfan et al. (2018), and Kibar (2021).

**TABLE 2:** Some morphological and nutritional properties of fresh *P. ostreatus* mushroom before storage.

Property	Mean ± SD	Property	Mean ± SD
Cap diameter (mm)	83.70 ± 18.31	Ash (%)	6.88 ± 0.71
Stem length (mm)	37.02 ± 8.82	TSS (%)	3.20 ± 0.27
Stem diameter (mm)	16.24 ± 2.89	Nitrogen (N, %)	4.36 ± 0.72
Average mushroom weight (g)	18.50 ± 3.23	Phosphorus (P, mg 100 g^−1^)	1544.28 ± 120.13
Dry matter (%)	11.14 ± 0.78	Potassium (K, mg 100 g^−1^)	3318.61 ± 328.65
L*	59.37 ± 3.40	Magnesium (Mg, mg 100 g^−1^)	198.57 ± 16.32
a*	7.27 ± 0.64	Calcium (Ca, mg 100 g^−1^)	196.82 ± 15.38
b*	16.38 ± 1.38	Iron (Fe, mg 100 g^−1^)	17.06 ± 2.37
c*	17.93 ± 1.49	Manganese (Mn, mg 100 g^−1^)	1.43 ± 0.11
h°	66.04 ± 0.94	Copper (Cu, mg 100 g^−1^)	1.40 ± 0.13
Protein (%)	27.25 ± 2.42	Zinc (Zn, mg 100 g^−1^)	8.13 ± 1.34
pH	5.36 ± 0.19		

Abbreviation: SD, Standard deviation.

### 3.2 Change of WL, Protein, Dry Matter, TSS, and pH Contents During Storage

The analysis of variance showed that the differences between storage periods in terms of WL, dry matter, and pH were statistically significant (*p* < 0.01). On the other hand, the effect of storage periods on protein and TSS was found to be insignificant. There were significant differences in terms of protein (*p* < 0.05) and TSS (*p* < 0.01) among the treatments. However, no statistically significant difference was found among the treatments in terms of WL, dry matter, and pH. The interaction between SP and T was highly significant (*p* < 0.01) in terms of all the examined parameters (WL, protein, dry matter, TSS, and pH), emphasizing the combined influence of storage period and treatments (Table 3).

**TABLE 3:** Effect of PUT, SA, CA, and MeJA on WL, protein, dry matter, TSS, and pH contents of *P. ostreatus* during storage at 4°C.

Storage period (SP)	WL (%)	Protein (%)	Dry matter (%)	TSS (%)	pH
Day 7	1.03 ± 0.12 b	27.40 ± 2.79 a	5.53 ± 0.28 a	1.86 ± 0.45	5.51 ± 0.11 b
Day 14	1.54 ± 0.21 a	27.80 ± 1.40 a	4.38 ± 0.50 b	2.04 ± 0.32	5.92 ± 0.24 a
**Treatment (T)**					
Control	1.25 ± 0.19 a	26.47 ± 2.22 ab	4.81 ± 0.95 a	1.63 ± 0.56 b	5.91 ± 0.45 a
0.5 mM PUT	1.32 ± 0.24 a	25.03 ± 0.26 b	4.95 ± 0.87 a	2.70 ± 0.37 a	5.61 ± 0.07 a
1.0 mM PUT	1.22 ± 0.24 a	28.22 ± 0.77 ab	4.21 ± 1.23 a	1.83 ± 0.10 b	5.62 ± 0.29 a
2.0 mM PUT	1.54 ± 0.50 a	26.94 ± 2.40 ab	5.08 ± 0.50 a	2.13 ± 0.54 ab	5.75 ± 0.17 a
0.5 mM SA	1.39 ± 0.27 a	28.69 ± 0.43 ab	5.33 ± 0.27 a	1.70 ± 0.48 b	5.71 ± 0.23 a
1.0 mM SA	1.29 ± 0.24 a	28.22 ± 0.79 ab	5.23 ± 0.57 a	2.00 ± 0.14 ab	5.71 ± 0.08 a
2.0 mM SA	1.35 ± 0.44 a	28.22 ± 0.77 ab	4.91 ± 0.27 a	1.68 ± 0.22 b	5.82 ± 0.27 a
0.5 mM CA	1.18 ± 0.25 a	27.88 ± 0.65 ab	5.31 ± 0.47 a	1.65 ± 0.26 b	5.56 ± 0.16 a
1.0 mM CA	1.18 ± 0.22 a	27.88 ± 2.03 ab	4.71 ± 0.80 a	1.93 ± 0.17 ab	5.74 ± 0.44 a
2.0 mM CA	1.16 ± 0.25 a	27.56 ± 1.40 ab	4.95 ± 0.73 a	2.15 ± 0.21 ab	5.76 ± 0.25 a
0.5 mM MeJA	1.23 ± 0.27 a	30.06 ± 3.86 a	5.04 ± 0.58 a	2.05 ± 0.13 ab	5.97 ± 0.52 a
1.0 mM MeJA	1.18 ± 0.24 a	28.81 ± 3.11 ab	4.82 ± 0.23 a	2.05 ± 0.24 ab	5.51 ± 0.19 a
2.0 mM MeJA	1.42 ± 0.40 a	24.84 ± 1.87 b	5.11 ± 1.15 a	1.90 ± 0.14 b	5.70 ± 0.05 a
**SP × T**					
Day 7 × Control	1.08 ± 0.08 efg	24.56 ± 0.35 ij	5.63 ± 0.04 bcd	1.20 ± 0.28 e	5.52 ± 0.03 k–o
Day 14 × Control	1.42 ± 0.03 bcd	28.38 ± 0.35 c–f	3.98 ± 0.03 o	2.05 ± 0.35 a–e	6.30 ± 0.03 ab
Day 7 × 0.5 mM PUT	1.11 ± 0.09 d–g	25.19 ± 0.27 hij	5.70 ± 0.07 bc	2.90 ± 0.42 a	5.57 ± 0.04 i–m
Day 14 × 0.5 mM PUT	1.53 ± 0.12 bc	24.88 ± 0.18 hij	4.20 ± 0.04 mn	2.50 ± 0.28 ab	5.65 ± 0.07 ijk
Day 7 × 1.0 mM PUT	1.00 ± 0.10 g	27.75 ± 0.37 c–g	5.27 ± 0.04 fg	1.75 ± 0.07 b–e	5.37 ± 0.04 mno
Day 14 × 1.0 mM PUT	1.43 ± 0.08 bcd	28.69 ± 0.98 cde	3.14 ± 0.06 p	1.90 ± 0.10 b–e	5.86 ± 0.06 d–h
Day 7 × 2.0 mM PUT	1.11 ± 0.11 d–g	24.88 ± 0.27 hij	5.51 ± 0.06 de	1.70 ± 0.14 b–e	5.61 ± 0.06 i–l
Day 14 × 2.0 mM PUT	1.97 ± 0.27 a	29.00 ± 0.39 cd	4.64 ± 0.08 kL	2.55 ± 0.35 ab	5.89 ± 0.03 d–g
Day 7 × 0.5 mM SA	1.14 ± 0.04 d–g	28.38 ± 0.39 c–f	5.56 ± 0.03 b–e	1.30 ± 0.24 de	5.51 ± 0.07 k–o
Day 14 × 0.5 mM SA	1.64 ± 0.04 abc	29.00 ± 0.18 cd	5.10 ± 0.04 gh	2.10 ± 0.14 a–d	5.90 ± 0.04 def
Day 7 × 1.0 mM SA	1.06 ± 0.04 fg	27.75 ± 0.35 c–g	5.72 ± 0.04 b	2.05 ± 0.07 a–e	5.65 ± 0.07 ijk
Day 14 × 1.0 mM SA	1.51 ± 0.05 bc	28.69 ± 0.82 cde	4.74 ± 0.03 jk	1.95 ± 0.21 b–e	5.77 ± 0.04 e–i
Day 7 × 2.0 mM SA	0.97 ± 0.02 g	28.69 ± 0.88 cde	5.14 ± 0.06 gh	1.85 ± 0.07 b–e	5.59 ± 0.04 i–l
Day 14 × 2.0 mM SA	1.72 ± 0.29 ab	27.75 ± 0.55 c–g	4.67 ± 0.04 kL	1.50 ± 0.14 cde	6.05 ± 0.07 cd
Day 7 × 0.5 mM CA	0.95 ± 0.03 g	28.38 ± 0.36 c–f	5.71 ± 0.03 bc	1.45 ± 0.21 cde	5.42 ± 0.03 L–o
Day 14 × 0.5 mM CA	1.41 ± 0.05 b–e	27.38 ± 0.35 d–g	4.90 ± 0.04 ij	1.85 ± 0.07 b–e	5.69 ± 0.04 g–k
Day 7 × 1.0 mM CA	0.98 ± 0.05 g	26.13 ± 0.18 ghi	5.40 ± 0.05 ef	1.90 ± 0.28 b–e	5.36 ± 0.06 no
Day 14 × 1.0 mM CA	1.38 ± 0.04 c–f	29.63 ± 0.45 bc	4.01 ± 0.03 o	1.95 ± 0.09 b–e	6.11 ± 0.06 bc
Day 7 × 2.0 mM CA	0.93 ± 0.04 g	26.44 ± 0.88 f–i	5.58 ± 0.03 bcd	2.00 ± 0.16 b–e	5.55 ± 0.07 j–n
Day 14 × 2.0 mM CA	1.39 ± 0.04 cde	28.69 ± 0.18 cde	4.31 ± 0.05 m	2.30 ± 0.14 abc	5.97 ± 0.04 cde
Day 7 × 0.5 mM MeJA	0.99 ± 0.02 g	33.38 ± 0.82 a	5.54 ± 0.06 cde	2.10 ± 0.14 a–d	5.52 ± 0.04 k–o
Day 14 × 0.5 mM MeJA	1.47 ± 0.09 bc	26.75 ± 0.35 e–h	4.54 ± 0.06 L	2.00 ± 0.15 b–e	6.42 ± 0.03 a
Day 7 × 1.0 mM MeJA	0.96 ± 0.05 g	31.50 ± 0.35 ab	5.01 ± 0.07 hi	2.05 ± 0.21 a–e	5.34 ± 0.06 o
Day 14 × 1.0 mM MeJA	1.40 ± 0.03 b–e	26.13 ± 0.18 ghi	4.62 ± 0.06 kL	2.05 ± 0.35 a–e	5.67 ± 0.04 h–k
Day 7 × 2.0 mM MeJA	1.13 ± 0.31 d–g	23.25 ± 0.53 j	6.11 ± 0.03 a	1.95 ± 0.21 b–e	5.67 ± 0.04 h–k
Day 14 × 2.0 mM MeJA	1.71 ± 0.22 abc	26.44 ± 0.27 f–i	4.11 ± 0.03 no	1.85 ± 0.07 b–e	5.73 ± 0.04 f–j
**Level of significance**					
SP	**	ns	**	ns	**
T	ns	*	ns	**	ns
SP × T	**	**	**	**	**

*Note:* Lowercase letters indicate differences in storage period, treatments and SP × T interaction.

Abbreviations: ±, Standard deviation of mean; CA, Citric acid; MeJA, Methyl jasmonate; ns, non‐significant; PUT, Putrescine; SA, Salicylic acid; TSS, Total soluble solids; WL, Weight loss.

* *p* < 0.05.

** *p* < 0.01.

When the effect of storage periods on WL was examined, the WL on the 14th day (1.54%) was found to be significantly higher than the WL on the 7th day (1.03%). It was determined that WL increased regularly during the storage period. WL remained comparable among treatments, with some treatments (e.g., 1.0 mM MeJA, 0.5–2.0 mM CA) showing slightly lower values. Depending on the postharvest treatments and storage periods considered in the study, WL values varied between 0.93% (Day 7 × 2.0 mM CA) and 1.97% (Day 14 × 2.0 mM PUT). It was determined that especially CA and MeJA were more effective in reducing WL (Table 3).

The losses in weight during storage of mushrooms are considered a parameter of quality decline (Khan et al. 2015). In addition to mushrooms having a thin epidermal layer that cannot prevent high transpiration rates, a high respiration rate causes an increase in WL during storage and a short shelf life (Cliffe‐Byrnes and O'Beirne 2008; Jiang et al. 2013). WL in mushrooms primarily occurs through transpiration and evaporation. Mushrooms are quite sensitive to rapid water loss. Water loss is a critical factor in reducing the storage life of fresh produce by accelerating deterioration during storage (Pan and Sasanatayart 2016). Consistent with our findings, previous studies have found that WL in different mushroom species increases regularly during storage (Jafri et al. 2013; Khan et al. 2015; Olotu et al. 2015; Gupta and Bhat 2016; Ventura‐Aguilar et al. 2017; Çavuşoğlu 2018). Villaescusa and Gil (2003) determined that the WL of *P. ostreatus* mushroom after 7 days of storage at 4°C varied between 1.1% and 5.0%. Compounds such as PUT, SA, CA, and MeJA are effective in reducing WL by preventing postharvest water loss and maintaining cellular integrity in mushrooms. PUT plays an important role in maintaining cellular membrane stability and water balance. SA prevents water evaporation by strengthening the cell wall. In addition, SA delays WL by inhibiting ethylene synthesis. CA prevents water loss by lowering pH and slows down the outflow of water from the cell by maintaining osmotic balance within the cell. Alali et al. (2023) stated that CA can reduce transpiration rates, close stomata, and minimize WL in fruits and vegetables. MeJA prevents water loss by protecting cell membranes from oxidative stress and helps maintain weight by reducing water loss in the fungus's tissues. Eleni and Theodoros (2011), Babu et al. (2014), and Rastegar et al. (2022) stated that PUT, CA, and SA have anti‐aging properties and are effective in reducing WL in vegetables by scavenging reactive oxygen species and preserving membrane integrity. In studies conducted on *P. florida* (Jafri et al. 2013), *P. ostreatus* (Ventura‐Aguilar et al. 2017), and *A. bisporus* (Sarıçam 2008; Khan et al. 2015; Gupta and Bhat 2016) mushrooms, it was found that postharvest CA applications significantly reduced WL compared to the control. Our findings are in line with previous studies. Olotu et al. (2015) reported that there was no significant difference between control and CA treatments in terms of WL in *P. ostreatus* mushroom after 30 days of storage at 4°C. In studies conducted on parsley (Üner 2018) and tomato (Davras et al. 2019), it was found that postharvest SA applications significantly reduced WL at the end of the storage period compared to the control. On the other hand, Akbulut (2015) reported that WL in tomato at the end of the storage period was higher in SA applications than in control. Our results are consistent with the researcher's findings. Gülsoylu (2015) reported that the effect of postharvest SA applications on WL in pepper was not significant. Çavuşoğlu (2018) stated that higher WL in *A. bisporus* mushroom was observed in MeJA applications at the end of the storage period compared to the control. On the other hand, studies conducted on cowpea (Fan et al. 2016a) and eggplant (Fan et al. 2016b; Yılmaz and Çavuşoğlu 2018) determined that postharvest MeJA applications significantly reduced WL compared to the control. In the study conducted by Jia et al. (2018) on cucumber, WL was found to be significantly lower in postharvest PUT application than in the control, and it was reported that WL could be reduced with PUT application. Kibar et al. (2023) found that SA, CA, and PUT applications significantly reduced the WL of broccoli compared to the control.

Among the treatments, the highest protein content was determined in 0.5 mM MeJA with 30.06%, while the lowest protein content was observed in 2.0 mM MeJA and 0.5 mM PUT (24.84% and 25.03%, respectively), which were not statistically different. When the SP × T interaction was examined, it was determined that the protein content ranged from 23.25% (Day 7 × 2.0 mM MeJA) to 33.38% (Day 7 × 0.5 mM MeJA) (Table 3).

Protein content is one of the most important components affecting mushroom quality and determining the nutritional value of mushrooms. Sulieman et al. (2019) stated that the most important feature that increases the nutritional value of mushrooms is their high protein content. It is reported that *P. ostreatus* mushroom is a good source of protein (17%–42%) (Khan et al. 2008; Akyüz and Kırbağ 2010). Decreases in the protein and sugar content of mushrooms are among the important indicators of spoilage in the postharvest period. Protein loss in mushrooms after harvest usually occurs due to metabolic processes, enzymatic activity, microbial degradation, and oxidative stress. PUT, SA, CA, and MeJA are effective compounds to prevent postharvest protein loss and preserve nutritional value in mushrooms. PUT, CA, and MeJA prevent protein loss by suppressing the activity of proteolytic enzymes that break down proteins. PUT regulates amino acid metabolism and ensures the preservation of protein content by preventing protein damage due to oxidative stress. SA activates enzymes involved in protein synthesis and prevents protein degradation by protecting cell membranes. CA maintains protein stability with its pH‐lowering effect and preserves protein content by increasing the activity of enzymes involved in protein metabolism. MeJA promotes protein synthesis by increasing stress tolerance. However, since high doses of some compounds may cause protein loss, the optimum dose needs to be determined. Motamedi et al. (2020) reported that PUT and CA applications significantly increased the protein content of *A. bisporus* mushroom compared to the control, and the highest protein content was found in 3 mM PUT application. Researchers also stated that the increase in protein content in postharvest polyamine applications may be due to the strong binding of polyamines to RNA, DNA, and proteins. Therefore, the results obtained in this current study are consistent with the findings of Motamedi et al. (2020). Contrary to our results, Ozturk et al. (2021) reported that significant increases in protein content were observed with the advancement of storage period in *C. cibarius* mushroom, and that postharvest CA application generally caused a decrease in protein content compared to the control. Sarıçam (2008) stated that the protein content of *A. bisporus* mushroom increased as the storage period progressed and that there was no statistically significant difference between CA and control applications in terms of protein amount during storage. Consistent with literature findings, although no statistically significant difference was observed in this study, increases in protein amount were detected during storage. On the other hand, Ramdas (2012) and Li et al. (2021) determined that protein content decreased as storage period increased in *Pleurotus* mushroom species. Kibar et al. (2023) reported that postharvest PUT, CA, and SA applications increased the protein content of broccoli compared to the control, which was compatible with our results.

The dry matter amount on the 7th day (5.53%) was found to be significantly higher than the dry matter amount on the 14th day (4.38%). The dry matter consistently decreased with the increase in the storage period. In the study, the minimum and maximum values in terms of dry matter amount in *P. ostreatus* mushroom, where different postharvest treatments and storage periods were applied, were determined in 1.0 mM PUT on the 14th day (3.14%) and in 2.0 mM MeJA on the 7th day (6.11%), respectively (Table 3).

In the postharvest process, water loss occurs as the metabolic activities of mushrooms continue, which can cause changes in the dry matter ratio. Since dry matter is used in respiration, it is an expected result that the amount of dry matter decreases during storage (Jiang et al. 2012). Compounds such as PUT, SA, CA, and MeJA can delay spoilage by maintaining or increasing the dry matter content in mushrooms. PUT prevents water loss by reducing the permeability of cell membranes and ensures the preservation of dry matter content. PUT also slows down transpiration and ensures that the dry matter ratio remains more stable. SA maintains cellular osmotic balance, prevents the dry matter content from decreasing by regulating intracellular water balance. CA maintains intracellular pH balance, prevents cellular water loss, and balances dry matter content. MeJA delays dry matter loss by increasing the activity of antioxidant enzymes. It also increases the dry matter ratio by preventing protein and sugar loss. Contrary to the results obtained in this study, Ozturk et al. (2021) stated that significant increases were observed in the dry matter amount of *C. cibarius* mushroom as the storage period progressed, and CA application generally caused a decrease in the amount of dry matter compared to the control. Kibar et al. (2024) found that PUT, SA, and CA applications increased the dry matter content of cauliflower compared to the control.

Among the treatments, the highest TSS was determined in the 0.5 mM PUT with 2.70%. The lowest TSS was detected in control, 1.0 mM PUT, 0.5 mM SA, 2.0 mM SA, 0.5 mM CA, and 2.0 mM MeJA, which were statistically no different among them. In terms of TSS, higher values were obtained from all postharvest treatments considered in the study compared to the control. TSS varied between 1.20% and 2.90% depending on the storage periods and postharvest treatments (Table 3).

TSS includes components of mushrooms such as sugar, organic acids, amino acids, and dissolved minerals and is evaluated as one of the quality parameters. A significant portion of the water‐soluble dry matter in mushrooms consists of sugars. During the postharvest period, water loss, metabolic changes, and enzymatic processes occurring in mushrooms can directly affect the TSS level. It is known that sugars are broken down during respiration and decrease during storage. In addition, sugars are oxidized by very fast metabolic activity and more losses occur as the storage period is extended (Ares et al. 2007). The increase in soluble solids of horticultural products during storage can generally be explained by the proportional increase in sugars in the fruit juice because of water loss. Jiang et al. (2012) reported that the amount of water‐soluble dry matter increased during storage in *Lentinus edodes* mushroom, and this increase was greater in applications where water loss was high. PUT, SA, CA, and MeJA can prevent quality loss by increasing or maintaining TSS content in mushrooms. PUT may positively affect TSS content by maintaining cellular stability and osmotic balance. SA can maintain or increase TSS levels by regulating sugar metabolism and delaying cellular degradation. CA stabilizes TSS content by regulating enzymatic activity and maintaining intracellular pH balance. MeJA preserves sugar content by preventing cellular water loss. Villaescusa and Gil (2003) determined that the TSS amount in *P. ostreatus* mushroom decreased compared to the initial value after 7 days of storage at 4°C, which was consistent with our findings. Motamedi et al. (2020) found that postharvest PUT and CA applications in *A. bisporus* mushroom increased the TSS value compared to the control. Therefore, the results obtained in our study are like those of Motamedi et al. (2020). Likewise, Jia et al. (2018) found that the amount of TSS in cucumber at the end of the storage period was significantly higher in PUT application than in the control. Jafri et al. (2013) reported that the TSS content of *P. florida* mushroom stored at 4°C for 25 days using CA solution increased during storage. Çavuşoğlu (2018) stated that the TSS content in *A. bisporus* mushroom stored at 4°C for 20 days decreased compared to the initial value at the end of the storage period, and lower TSS values were observed in MeJA applications compared to the control. It was determined that postharvest MeJA application had a positive effect on TSS in cowpea and eggplant (Fan et al. 2016a; Yılmaz and Çavuşoğlu 2018). In studies conducted on tomato and pepper, it was reported that TSS content was higher in SA applications than in the control at the end of the storage period (Akbulut 2015; Gülsoylu 2015). Our findings are parallel with these results.

The pH also increased significantly as the storage period increased, reflecting changes in the biochemical environment during storage. The highest pH value (6.42) was determined on the 14th day in 0.5 mM MeJA, while the lowest pH value (5.34) was observed on the 7th day in 1.0 mM MeJA (Table 3).

The pH value is of critical importance in the biochemical processes of mushrooms. The pH value of mushrooms is an important quality indicator that affects cellular metabolism, enzyme activity, and microbial stability. Appropriate pH conditions can limit the growth of harmful microorganisms and thus prevent the mushroom from spoiling and ensure that its nutritional value is preserved for a long time. During the postharvest process, the pH value of mushrooms may change due to water loss, organic acid changes, microbial activity, and metabolic processes. An increase in pH value may be an indicator of rapid spoilage and microbial growth. Compounds such as PUT, SA, CA, and MeJA can help prevent postharvest quality loss by regulating pH levels in mushrooms. PUT can control pH changes by stabilizing cell membranes and regulating intracellular ion balance. SA may help maintain pH stability by regulating enzyme activity and activating antioxidant systems. In addition, SA delays cellular degradation by suppressing ethylene synthesis and slows microbial growth. CA limits microbial activity by lowering pH levels and maintains ionic balance by increasing cellular stability. MeJA maintains pH balance by reducing oxidative stress and optimizes ion balance by maintaining cellular membrane integrity. However, determining the application dose correctly is critical to prevent excessive pH elevation or decrease. Like the results obtained in this study, Villaescusa and Gil (2003) determined that the pH value of *P. ostreatus* mushroom increased compared to the initial value after 7 days of storage at 4°C. Similarly, it was stated that the pH value of *A. bisporus* mushroom stored at 4°C for 20 days increased compared to the initial value in MeJA applications at the end of the storage period (Çavuşoğlu 2018). On the other hand, Jafri et al. (2013) reported that the pH of *P. florida* mushroom stored at 4°C for 25 days using CA solution decreased during storage. Researchers also stated that the lower pH in samples treated with CA was due to the acidic effect of CA. In studies conducted on pepper and tomato, it was determined that the pH value in SA applications was lower than the control at the end of the storage period (Gülsoylu 2015; Davras et al. 2019). In another study, it was determined that the effect of postharvest MeJA applications on pH in eggplant was not significant (Yılmaz and Çavuşoğlu 2018). Likewise, the effect of PUT, SA, and CA treatments on the pH value of broccoli was found to be insignificant (Kibar et al. 2023). The results obtained in this study were found to be consistent with previous studies.

### 3.3 Change of Ash and Mineral Element Contents During Storage

The storage period had no significant effect on ash, N, K, and Mg contents, indicating that these parameters remained relatively stable between Day 7 and Day 14. However, a significant effect of storage period on P content was observed (*p* < 0.05). Treatments significantly influenced N (*p* < 0.05), K (*p* < 0.01), and P (*p* < 0.01). On the other hand, no significant effect of treatments was observed in terms of ash and Mg contents. The SP × T interaction in terms of the nutritional properties mentioned in Table 4, except for Mg content, was found to be statistically significant (*p* < 0.01).

**TABLE 4:** Effect of PUT, SA, CA, and MeJA on ash, N, K, P, and Mg contents of *P. ostreatus* during storage at 4°C.

Storage period (SP)	Ash (%)	*N* (%)	K (mg 100 g^−1^)	P (mg 100 g^−1^)	Mg (mg 100 g^−1^)
Day 7	6.97 ± 0.89 a	4.38 ± 0.45 a	3249.08 ± 427.72 a	1524.08 ± 166.38 b	190.08 ± 18.88 a
Day 14	7.45 ± 1.49 a	4.45 ± 0.22 a	3161.00 ± 248.92 a	1615.31 ± 136.20 a	190.62 ± 12.61 a
**Treatment (T)**					
Control	5.95 ± 0.28 a	4.24 ± 0.36 ab	2740.50 ± 405.13 b	1345.00 ± 98.56 b	175.40 ± 20.72 a
0.5 mM PUT	7.43 ± 1.16 a	4.01 ± 0.14 b	2924.00 ± 146.46 ab	1317.00 ± 79.36 b	175.30 ± 18.30 a
1.0 mM PUT	7.02 ± 0.99 a	4.52 ± 0.12 ab	2890.50 ± 174.25 ab	1492.00 ± 24.40 ab	181.45 ± 11.55 a
2.0 mM PUT	6.99 ± 1.10 a	4.31 ± 0.38 ab	3161.50 ± 409.64 ab	1496.50 ± 68.55 ab	183.05 ± 14.13 a
0.5 mM SA	7.41 ± 0.62 a	4.59 ± 0.17 ab	3421.50 ± 126.95 ab	1611.50 ± 43.82 a	197.10 ± 6.13 a
1.0 mM SA	7.93 ± 0.79 a	4.52 ± 0.12 ab	3576.50 ± 130.04 a	1602.00 ± 52.10 a	198.35 ± 9.70 a
2.0 mM SA	6.46 ± 0.75 a	4.52 ± 0.12 ab	3381.00 ± 525.48 ab	1696.00 ± 43.30 a	206.65 ± 16.79 a
0.5 mM CA	6.94 ± 0.83 a	4.46 ± 0.10 ab	3192.00 ± 243.17 ab	1555.50 ± 33.77 ab	191.60 ± 12.81 a
1.0 mM CA	7.90 ± 1.57 a	4.46 ± 0.33 ab	3273.00 ± 494.35 ab	1728.00 ± 34.28 a	201.70 ± 11.99 a
2.0 mM CA	6.95 ± 0.13 a	4.41 ± 0.22 ab	3285.00 ± 256.99 ab	1566.00 ± 103.17 ab	188.45 ± 11.62 a
0.5 mM MeJA	7.06 ± 0.15 a	4.81 ± 0.62 a	3513.00 ± 170.53 a	1707.50 ± 36.74 a	193.85 ± 17.04 a
1.0 mM MeJA	7.71 ± 2.07 a	4.61 ± 0.50 ab	3313.50 ± 116.68 ab	1548.00 ± 36.04 ab	190.90 ± 12.31 a
2.0 mM MeJA	8.04 ± 2.75 a	3.98 ± 0.30 b	2993.50 ± 175.25 ab	1741.00 ± 144.50 a	190.70 ± 20.66 a
**SP × T**					
Day 7 × Control	6.19 ± 0.03 l	3.93 ± 0.06 ij	2390.00 ± 54.14 g	1174.00 ± 28.28 L	160.50 ± 16.22 a
Day 14 × Control	5.71 ± 0.08 no	4.54 ± 0.06 c–f	3091.00 ± 77.28 def	1516.00 ± 22.63 g–j	190.30 ± 16.78 a
Day 7 × 0.5 mM PUT	6.43 ± 0.04 jk	4.03 ± 0.04 hij	2887.00 ± 35.14 f	1249.00 ± 14.14 l	176.40 ± 28.28 a
Day 14 × 0.5 mM PUT	8.43 ± 0.04 d	3.98 ± 0.03 hij	2961.00 ± 53.28 f	1385.00 ± 17.14 k	174.20 ± 15.17 a
Day 7 × 1.0 mM PUT	7.88 ± 0.03 e	4.44 ± 0.06 c–g	2832.00 ± 45.26 f	1483.00 ± 19.90 hij	181.40 ± 17.14 a
Day 14 × 1.0 mM PUT	6.16 ± 0.08 L	4.59 ± 0.14 cde	2949.00 ± 28.28 f	1501.00 ± 14.14 g–j	181.50 ± 19.57 a
Day 7 × 2.0 mM PUT	7.94 ± 0.06 e	3.98 ± 0.04 hij	2814.00 ± 19.80 f	1439.00 ± 28.28j k	176.00 ± 19.13 a
Day 14 × 2.0 mM PUT	6.03 ± 0.04 lm	4.64 ± 0.06 cd	3509.00 ± 141.42 bc	1554.00 ± 18.49 ghi	190.10 ± 31.47 a
Day 7 × 0.5 mM SA	7.94 ± 0.09 e	4.54 ± 0.06 c–f	3343.00 ± 60.81 cd	1577.00 ± 38.28 efg	193.40 ± 12.83 a
Day 14 × 0.5 mM SA	6.87 ± 0.05 i	4.64 ± 0.03 cd	3500.00 ± 160.42 bc	1646.00 ± 24.14 def	200.80 ± 23.15 a
Day 7 × 1.0 mM SA	8.61 ± 0.06 d	4.44 ± 0.06 c–g	3688.00 ± 66.14 ab	1646.00 ± 14.14 def	201.90 ± 24.18 a
Day 14 × 1.0 mM SA	7.24 ± 0.06 g	4.59 ± 0.14 cde	3465.00 ± 47.28 bc	1558.00 ± 32.14 ghi	194.80 ± 25.66 a
Day 7 × 2.0 mM SA	7.11 ± 0.05 gh	4.59 ± 0.14 cde	3835.00 ± 49.50 a	1730.00 ± 48.28 bc	217.20 ± 42.46 a
Day 14 × 2.0 mM SA	5.81 ± 0.07 no	4.44 ± 0.06 c–g	2927.00 ± 38.18 f	1662.00 ± 42.14 bcd	196.10 ± 36.88 a
Day 7 × 0.5 mM CA	6.22 ± 0.07 kL	4.54 ± 0.06 c–f	3402.00 ± 48.28 c	1549.00 ± 24.14 ghi	196.40 ± 16.85 a
Day 14 × 0.5 mM CA	7.66 ± 0.06 f	4.38 ± 0.06 d–g	2982.00 ± 64.14 f	1562.00 ± 55.14 gh	186.80 ± 28.14 a
Day 7 × 1.0 mM CA	6.54 ± 0.06 j	4.18 ± 0.03 ghi	3695.00 ± 28.28 ab	1711.00 ± 12.73 bcd	204.50 ± 15.53 a
Day 14 × 1.0 mM CA	9.25 ± 0.07 c	4.74 ± 0.06 bc	2851.00 ± 144.28 f	1745.00 ± 21.21 b	198.90 ± 34.48 a
Day 7 × 2.0 mM CA	7.05 ± 0.07 ghi	4.23 ± 0.14 f–i	3507.00 ± 84.14 bc	1477.00 ± 32.14 ij	189.60 ± 18.47 a
Day 14 × 2.0 mM CA	6.84 ± 0.06 i	4.59 ± 0.03 cde	3063.00 ± 38.28 def	1655.00 ± 47.07 cde	187.30 ± 18.61 a
Day 7 × 0.5 mM MeJA	7.18 ± 0.08 g	5.34 ± 0.14 a	3572.00 ± 57.28 abc	1718.00 ± 16.14 bcd	204.70 ± 26.33 a
Day 14 × 0.5 mM MeJA	6.93 ± 0.04 hi	4.28 ± 0.06 e–h	3454.00 ± 26.14 bc	1697.00 ± 26.14 bcd	183.00 ± 19.27 a
Day 7 × 1.0 mM MeJA	5.91 ± 0.04 mn	5.04 ± 0.06 ab	3328.00 ± 141.42 cd	1530.00 ± 42.43 ghi	194.60 ± 28.61 a
Day 14 × 1.0 mM MeJA	9.50 ± 0.07 b	4.18 ± 0.03 ghi	3299.00 ± 132.42 cde	1530.00 ± 21.21 ghi	187.20 ± 33.14 a
Day 7 × 2.0 mM MeJA	5.66 ± 0.03 o	3.72 ± 0.08 j	2945.00 ± 63.40 f	1566.00 ± 48.28 fgh	174.40 ± 21.43 a
Day 14 × 2.0 mM MeJA	10.42 ± 0.04 a	4.23 ± 0.04 f–i	3042.00 ± 59.40 ef	1952.00 ± 58.28 a	207.00 ± 24.29 a
**Level of significance**					
SP	ns	ns	ns	*	ns
T	ns	*	**	**	ns
SP × T	**	**	**	**	ns

*Note:* Lowercase letters indicate differences in storage period, treatments and SP × T interaction.

Abbreviations: ±, Standard deviation of mean; CA, Citric acid; K, Potassium; MeJA, Methyl jasmonate; Mg, Magnesium; N, Nitrogen; ns, non‐significant; P, Phosphorus; PUT, Putrescine; SA, Salicylic acid.

* *p* < 0.05.

** *p* < 0.01.

When the SP × T interaction was examined, the ash content varied between 5.66% and 10.42%. The highest ash content was observed in 2.0 mM MeJA on the 14th day, while the lowest ash content was determined in 2.0 mM MeJA on the 7th day (Table 4).

The ash content of mushrooms primarily comprises essential minerals, including K, P, Mg, and Ca. The change in the ash content of mushrooms during the storage period directly affects the change in the mineral content (Ramdas 2012). The rise in ash content during the storage period is likely attributed to moisture loss. As in our study, Ozturk et al. (2021) stated that increases in the amount of ash were observed as a natural result of water loss with the advancement of the storage period in *C. cibarius* mushroom. Researchers also reported that the ash content at the end of the storage period was lower in CA application compared to the control. On the other hand, Ramdas (2012) determined that the ash content of *Pleurotus* mushroom species stored at different storage temperatures decreased as the storage period increased. Kibar et al. (2023) reported that there was no significant difference in terms of the ash content of broccoli among SA, CA, PUT, and control applications during storage. In another study, it was found that PUT, SA, and CA applications significantly increased the ash content of cauliflower compared to the control (Kibar et al. 2024). CA may contribute to a higher ash content by chelating metal ions and increasing their solubility (Sánchez‐Moreno et al. 1998).

Among the treatments, the highest N content was determined in 0.5 mM MeJA with 4.81%, while the lowest N content was found in 2.0 mM MeJA and 0.5 mM PUT (3.98% and 4.01%, respectively), which were not statistically different from each other. In the study, it was determined that N content varied between 3.72% (Day 7 × 2.0 mM MeJA) and 5.34% (Day 7 × 0.5 mM MeJA). 0.5 mM MeJA was particularly effective in maintaining N levels (Table 4).

1.0 mM SA and 0.5 mM MeJA treatments showed the highest K contents. Conversely, the control had the lowest K content. All postharvest treatments considered in the study significantly increased K content compared to the control. When the SP × T interaction was examined, the highest K content was determined in 2.0 mM SA on day 7, while the lowest K content was observed in the control on day 7 (Table 4).

P content on day 14 (1615.31 mg 100 g^−1^) was found to be significantly higher than the P content on day 7 (1524.08 mg 100 g^−1^). P content increased significantly during storage, which was probably due to postharvest biochemical changes or differential nutrient mobilization. 0.5 mM SA, 1.0 mM SA, 2.0 mM SA, 1.0 mM CA, 0.5 mM MeJA, and 2.0 mM MeJA treatments had the highest P contents. On the other hand, the lowest P contents were determined in control and 0.5 mM PUT. In terms of P content, higher values were obtained than the control in all postharvest treatments considered in the study, except for 0.5 mM PUT. P content in *P. ostreatus* mushroom, where different postharvest treatments and storage periods were applied, varied between 1174.00 mg 100 g^−1^ and 1952.00 mg 100 g^−1^. The highest P content was determined in 2.0 mM MeJA on the 14th day, while the lowest P content was recorded in control and 0.5 mM PUT on the 7th day (Table 4).

Mg content of *P. ostreatus* appears to be relatively resilient to external factors such as storage period and treatment (Table 4).

As seen in Table 5, there were statistically significant differences between storage periods for Fe (*p* < 0.05) and Zn (*p* < 0.01) contents, while no statistically significant difference was found between storage periods in terms of Ca, Mn, and Cu contents. The effects of treatments on Ca (*p* < 0.01), Mn (*p* < 0.05), Cu (*p* < 0.01), and Zn (*p* < 0.05) contents of *P. ostreatus* were statistically significant. However, the effect of treatments on Fe content was found to be insignificant. The SP × T interaction in terms of all minerals mentioned in Table 5 was found to be statistically significant (*p* < 0.01).

**TABLE 5:** Effect of PUT, SA, CA, and MeJA on Ca, Fe, Mn, Cu, and Zn contents of *P. ostreatus* during storage at 4°C.

Storage period (SP)	Ca (mg 100 g^−1^)	Fe (mg 100 g^−1^)	Mn (mg 100 g^−1^)	Cu (mg 100 g^−1^)	Zn (mg 100 g^−1^)
Day 7	184.62 ± 32.98 a	17.34 ± 3.40 b	1.40 ± 0.10 a	1.46 ± 1.39 a	7.48 ± 1.21 b
Day 14	144.52 ± 33.06 a	23.64 ± 4.71 a	1.59 ± 0.59 a	1.47 ± 0.96 a	9.38 ± 2.04 a
**Treatment (T)**					
Control	131.20 ± 17.74 bc	13.97 ± 0.47 a	1.42 ± 0.07 b	2.66 ± 2.08 ab	7.00 ± 0.73 b
0.5 mM PUT	145.20 ± 36.23 bc	14.70 ± 0.54 a	1.38 ± 0.17 b	1.27 ± 0.43 ab	7.00 ± 1.91 b
1.0 mM PUT	101.22 ± 28.68 c	17.64 ± 2.95 a	1.39 ± 0.09 b	0.95 ± 0.10 b	7.78 ± 0.55 ab
2.0 mM PUT	100.03 ± 14.73 c	21.80 ± 1.43 a	1.38 ± 0.07 b	0.98 ± 0.09 b	7.91 ± 0.09 ab
0.5 mM SA	288.10 ± 23.07 ab	22.77 ± 0.80 a	1.57 ± 0.08 ab	0.82 ± 0.07 b	7.65 ± 0.13 ab
1.0 mM SA	192.75 ± 22.28 bc	18.65 ± 3.41 a	1.45 ± 0.14 b	1.12 ± 0.04 ab	8.75 ± 2.39 ab
2.0 mM SA	100.50 ± 8.50 c	20.17 ± 2.85 a	1.44 ± 0.05 b	3.37 ± 1.56 a	10.32 ± 3.67 a
0.5 mM CA	94.63 ± 21.30 c	19.89 ± 3.05 a	1.30 ± 0.09 b	0.88 ± 0.09 b	8.13 ± 1.39 ab
1.0 mM CA	95.01 ± 14.21 c	16.80 ± 0.77 a	1.43 ± 0.06 b	0.91 ± 0.43 b	9.80 ± 0.71 ab
2.0 mM CA	102.47 ± 20.80 c	15.93 ± 0.30 a	1.42 ± 0.08 b	2.99 ± 1.71 ab	10.99 ± 2.71 a
0.5 mM MeJA	400.45 ± 30.80 a	34.72 ± 3.97 a	1.45 ± 0.09 b	1.34 ± 0.71 ab	9.15 ± 0.68 ab
1.0 mM MeJA	102.64 ± 23.06 c	16.50 ± 4.05 a	1.42 ± 0.10 b	0.76 ± 0.29 b	7.38 ± 0.68 ab
2.0 mM MeJA	285.25 ± 33.74 ab	32.87 ± 3.94 a	2.41 ± 0.31 a	0.97 ± 0.22 b	7.74 ± 1.25 ab
**SP × T**					
Day 7 × Control	146.30 ± 2.42 fgh	14.30 ± 0.42 mn	1.46 ± 0.06 b–f	0.86 ± 0.06 ghi	6.37 ± 0.04 p
Day 14 × Control	116.10 ± 5.66 g–k	13.63 ± 0.14 o	1.38 ± 0.06 b–f	4.46 ± 0.06 a	7.63 ± 0.04 jkl
Day 7 × 0.5 mM PUT	176.50 ± 2.71 ef	15.16 ± 0.12 kL	1.23 ± 0.04 ef	1.64 ± 0.06 c	5.34 ± 0.06 q
Day 14 × 0.5 mM PUT	113.90 ± 4.24 h–l	14.23 ± 0.09 mn	1.52 ± 0.06 bcd	0.89 ± 0.04 f–i	8.65 ± 0.07 f
Day 7 × 1.0 mM PUT	76.63 ± 4.04 m	15.08 ± 0.15 L	1.40 ± 0.14 b–f	0.99 ± 0.04 e–i	8.25 ± 0.07 gh
Day 14 × 1.0 mM PUT	125.80 ± 7.07 ghi	20.19 ± 0.22 h	1.37 ± 0.04 b–f	0.90 ± 0.14 f–i	7.31 ± 0.13 lm
Day 7 × 2.0 mM PUT	87.35 ± 3.07 j–m	23.03 ± 0.41 de	1.35 ± 0.09 b–f	0.91 ± 0.07 f–i	7.94 ± 0.06 hij
Day 14 × 2.0 mM PUT	112.70 ± 2.83 h–m	20.56 ± 0.16 h	1.41 ± 0.07 b–f	1.05 ± 0.05 e–h	7.88 ± 0.16 ijk
Day 7 × 0.5 mM SA	302.20 ± 1.41 c	23.45 ± 0.14 d	1.51 ± 0.07 b–e	0.76 ± 0.06 hij	7.60 ± 0.14 kL
Day 14 × 0.5 mM SA	274.00 ± 28.28 c	22.08 ± 0.20 fg	1.63 ± 0.04 b	0.87 ± 0.03 f–i	7.69 ± 0.14 ijk
Day 7 × 1.0 mM SA	192.80 ± 2.76 de	15.70 ± 0.28 jk	1.34 ± 0.06 c–f	1.11 ± 0.06 efg	6.68 ± 0.07 op
Day 14 × 1.0 mM SA	192.70 ± 2.82 de	21.60 ± 0.16 g	1.56 ± 0.06 bc	1.13 ± 0.04 efg	10.82 ± 0.11 b
Day 7 × 2.0 mM SA	95.10 ± 7.07 i–m	22.63 ± 0.14 ef	1.42 ± 0.04 b–f	4.72 ± 0.06 a	7.14 ± 0.08 mn
Day 14 × 2.0 mM SA	105.90 ± 7.07 i–m	17.70 ± 0.14 i	1.46 ± 0.06 b–f	2.02 ± 0.14 b	13.50 ± 0.14 a
Day 7 × 0.5 mM CA	77.65 ± 2.83 lm	25.13 ± 0.19 c	1.37 ± 0.03 b–f	0.89 ± 0.04 f–i	6.93 ± 0.04 no
Day 14 × 0.5 mM CA	111.60 ± 14.14 h–m	14.65 ± 0.11 lm	1.22 ± 0.06 f	0.87 ± 0.14 f–i	9.33 ± 0.04 e
Day 7 × 1.0 mM CA	83.11 ± 2.83 klm	16.13 ± 0.12 j	1.47 ± 0.04 b–f	0.54 ± 0.06 j	9.18 ± 0.03 e
Day 14 × 1.0 mM CA	106.90 ± 15.66 i–m	17.46 ± 0.17 i	1.39 ± 0.04 b–f	1.28 ± 0.06 de	10.41 ± 0.06 c
Day 7 × 2.0 mM CA	86.04 ± 4.24 j–m	15.67 ± 0.15 jk	1.48 ± 0.05 b–f	4.47 ± 0.04 a	8.64 ± 0.06 f
Day 14 × 2.0 mM CA	118.90 ± 24.14 g–k	16.18 ± 0.03 j	1.36 ± 0.08 b–f	1.51 ± 0.08 cd	13.34 ± 0.08 a
Day 7 × 0.5 mM MeJA	574.20 ± 2.83 a	13.96 ± 0.17 no	1.46 ± 0.06 b–f	0.73 ± 0.03 ij	9.73 ± 0.04 d
Day 14 × 0.5 mM MeJA	226.70 ± 16.14 d	55.47 ± 0.14 a	1.43 ± 0.16 b–f	1.95 ± 0.07 b	8.56 ± 0.07 fg
Day 7 × 1.0 mM MeJA	83.98 ± 1.41 klm	12.99 ± 0.14 p	1.45 ± 0.07 b–f	0.52 ± 0.06 j	6.79 ± 0.05 o
Day 14 × 1.0 mM MeJA	121.30 ± 14.14 g–j	20.01 ± 0.17 h	1.39 ± 0.14 b–f	0.99 ± 0.14 e–i	7.96 ± 0.06 hi
Day 7 × 2.0 mM MeJA	418.20 ± 2.83 b	12.13 ± 0.17 q	1.27 ± 0.04 def	0.78 ± 0.09 hij	6.66 ± 0.09 op
Day 14 × 2.0 mM MeJA	152.30 ± 22.14 fg	53.60 ± 0.18 b	3.54 ± 0.08 a	1.16 ± 0.07 ef	8.82 ± 0.09 f
**Level of significance**					
SP	ns	*	ns	ns	**
T	**	ns	*	**	*
SP × T	**	**	**	**	**

*Note:* Lowercase letters indicate differences in storage period, treatments and SP × T interaction.

Abbreviations: ±, Standard deviation of mean; Ca, Calcium; CA, Citric acid; Cu, Copper; Fe, Iron; MeJA, Methyl jasmonate; Mn, Manganese; ns, non‐significant; PUT, Putrescine; SA, Salicylic acid; Zn, Zinc.

* *p* < 0.05.

** *p* < 0.01.

Among the treatments, the highest Ca content was found in 0.5 mM MeJA with 400.45 mg 100 g^−1^. On the other hand, 1.0 mM PUT, 2.0 mM PUT, 2.0 mM SA, 0.5 mM CA, 1.0 mM CA, and 2.0 mM CA treatments were statistically in the same group and were found to have the lowest calcium content. Ca content ranged from 76.63 mg 100 g^−1^ to 574.20 mg 100 g^−1^ depending on the storage periods and postharvest treatments. On day 7, 0.5 mM MeJA treatment showed the highest Ca value. This indicates that SP × T interaction plays critical roles in modulating calcium levels (Table 5).

Iron content significantly increased during storage, rising from 17.34 mg 100 g^−1^ (Day 7) to 23.64 mg 100 g^−1^ (Day 14). When the SP × T interaction was examined, Fe content varied between 12.13 mg 100 g^−1^ and 55.47 mg 100 g^−1^. On day 14, 0.5 mM MeJA treatment had the highest Fe content, indicating that MeJA is particularly effective when combined with extended storage (Table 5).

Treatments significantly influenced Mn content. 2.0 mM MeJA possessed the highest Mn value (2.41 mg 100 g^−1^). It was closely followed by 0.5 mM SA treatment. On the other hand, all treatments except 2.0 mM MeJA and 0.5 mM SA were statistically in the same group and were found to have the lowest Mn content. When the SP × T interaction was examined, the highest Mn content was determined in 2.0 mM MeJA on the 14th day, while the lowest Mn content was observed in 0.5 mM CA on the 14th day (Table 5).

Cu content was significantly affected by the treatments. The highest Cu content was determined in 2.0 mM SA (3.37 mg 100 g^−1^). On the contrary, the lowest Cu content was recorded in 1.0 mM PUT, 2.0 mM PUT, 0.5 mM SA, 0.5 mM CA, 1.0 mM CA, 1.0 mM MeJA, and 2.0 mM MeJA treatments. In the study, Cu content varied between 0.52 mg 100 g^−1^ and 4.72 mg 100 g^−1^ depending on the storage period and treatments (Table 5).

When the effect of storage periods on Zn content was examined, the Zn content on the 14th day (9.38 mg 100 g^−1^) was found to be significantly higher than the Zn content on the 7th day (7.48 mg 100 g^−1^). As the storage period increased, the Zn content also significantly increased. Among the treatments, the highest Zn content was determined in 2.0 mM SA and 2.0 mM CA treatments (10.32 mg 100 g^−1^ and 10.99 mg 100 g^−1^, respectively), while the lowest Zn content (7.00 mg 100 g^−1^) was recorded in control and 0.5 mM PUT. Except for 0.5 mM PUT, all postharvest treatments considered in the study significantly increased the Zn content compared to the control. When the SP × T interaction was examined, the highest Zn content was determined in 2.0 mM SA and 2.0 mM CA on the 14th day, while the lowest Zn content was observed in 0.5 mM PUT on the 7th day (Table 5).

Storage duration generally resulted in increased levels of P, Fe, and Zn, while other minerals remained relatively stable. The results demonstrate that specific treatments, particularly MeJA and SA, effectively enhance mineral content in *P. ostreatus* during storage.

Kalač (2009) reported that the most abundant minerals in mushrooms are K, Ca, Mg, Na, P, and S. *Pleurotus* mushroom species are important sources of mineral elements (Adebayo and Oloke 2017). Mineral element contents of mushrooms may vary depending on postharvest storage methods. During the postharvest process, mineral element content usually decreases due to increased respiration and oxidative stress. Kibar (2021) determined that essential mineral contents such as K, Mg, P, Ca, Fe, Zn, and Mn decreased in *P. ostreatus* mushroom after cold storage applications compared to the fresh mushroom sample. The rise in ash content during the storage period results in a higher concentration of minerals in the product.

Similar to the findings obtained in this study, in the study conducted by Motamedi et al. (2020), it was determined that PUT and CA applications applied in the postharvest period in *A. bisporus* mushroom significantly affected the macro and micro element contents of the mushroom. While there was no difference between the control and PUT applications in terms of Ca content, significantly higher Ca values were obtained from the CA application compared to the control. Significantly higher values were obtained from the PUT and CA applications compared to the control in terms of Fe and K contents. It was reported that PUT application caused an increase in the nutritional value of the mushroom. The researchers also stated that the increased intake of minerals such as N, P, and K with PUT application may be because of the role of polyamines on many biochemical and physiological processes. Coşkuner and Özdemir (2000) compared the mineral element contents in fresh and CA‐blanched *A. bisporus* mushroom and reported that blanching with CA slightly reduced Fe, Cu, Mn, and Zn contents compared to fresh mushrooms, but these decreases were not statistically significant. Kibar et al. (2023) reported that significant differences among CA, SA, PUT, and control treatments were found in terms of P, K, and K contents of broccoli, while there was no significant difference in terms of Mg content. The researchers also found that CA, SA, and PUT applications increased P and Ca contents of broccoli and decreased K content compared to the control. In another study, Kibar et al. (2024) determined that the effects of postharvest PUT, SA, and CA applications on P, K, Mg, Ca, Fe, Mn, Zn, and Cu contents of cauliflower were significant, and PUT, SA, and CA applications increased mineral element contents compared to the control. The efficacy of postharvest chemical applications can vary based on genotype, application dose, laboratory conditions, and storage conditions (Assar et al. 2012).

In the present study, the mineral contents examined were tried to be protected with PUT, SA, CA, and MeJA applications. These applications were found to be generally effective compared to the control. Biochemical regulators such as PUT, SA, CA, and MeJA can reduce nutrient loss and maintain mineral balance in mushrooms. These compounds may affect the physiological processes that regulate the uptake and transport of mineral elements in mushrooms after harvest. CA can increase the bioavailability of trace elements such as Fe, Zn, and Mn by increasing the solubility of mineral ions and optimizing the mineral balance of mushrooms by adjusting the pH balance. SA can increase Ca and Mg transport in fungal cells. PUT and SA may preserve K levels by increasing cell membrane stability. MeJA and SA may help preserve important minerals such as Ca, K, and Mg by reducing lipid peroxidation. Both MeJA and SA contribute to improving postharvest nutritional quality and consumer acceptance by increasing the accumulation of bioactive compounds such as anthocyanins, flavonoids, and phenolic acids (Asghari and Hasanlooe 2015; Asghari 2019). Depending on the application dose, storage period, and mushroom species, the effects of these compounds on the mineral contents of the mushroom may vary.

In studies conducted on different mushroom species, it was determined that postharvest PUT, SA, CA, and MeJA applications had positive effects on extending the shelf life and preserving postharvest quality (Olotu et al. 2015; Dokhanieh and Aghdam 2016; Gupta and Bhat 2016; Ventura‐Aguilar et al. 2017; Motamedi et al. 2020). The results obtained in the present study are generally consistent with the findings reported in previous studies.

### 3.4 Change of Mushroom Color During Storage

Table 6 presents data on how different treatments (PUT, SA, CA, and MeJA) and storage periods (7 days and 14 days) affect the color properties of *P. ostreatus* stored at 4°C. The storage period significantly influenced the color properties (*p* < 0.01) except for Hue angle. Significant differences (*p* < 0.01) were observed among treatments in terms of L* and Hue angle color values. On the other hand, no significant effect of treatments on a*, b*, and Chroma values was detected. The SP × T interaction in terms of L* (*p* < 0.01), a* (*p* < 0.01), b* (*p* < 0.01), chroma (*p* < 0.05), and hue angle (*p* < 0.01) color values was found to be statistically significant.

**TABLE 6:** Effect of PUT, SA, CA, and MeJA on color properties of *P. ostreatus* during storage at 4°C.

Storage period (SP)	L*	a*	b*	Chroma	Hue angle
Day 7	48.80 ± 3.87 b	6.37 ± 0.83 b	15.54 ± 1.29 b	16.78 ± 1.39 b	67.72 ± 2.04 a
Day 14	50.85 ± 5.32 a	7.31 ± 1.43 a	17.39 ± 2.74 a	19.46 ± 2.80 a	67.34 ± 2.44 a
**Treatment (T)**					
Control	50.06 ± 4.85 ab	6.42 ± 1.07 a	15.62 ± 2.39 a	16.89 ± 2.57 a	67.69 ± 1.75 ab
0.5 mM PUT	50.89 ± 5.23 a	6.92 ± 1.04 a	16.51 ± 2.81 a	17.90 ± 2.95 a	66.85 ± 1.70 bc
1.0 mM PUT	48.01 ± 4.34 ab	6.39 ± 0.78 a	15.73 ± 1.58 a	16.99 ± 1.65 a	67.90 ± 2.03 ab
2.0 mM PUT	50.34 ± 4.26 ab	6.82 ± 1.55 a	16.83 ± 3.09 a	18.18 ± 3.39 a	68.28 ± 1.77 ab
0.5 mM SA	49.70 ± 6.03 ab	7.86 ± 1.93 a	17.84 ± 3.23 a	19.51 ± 3.71 a	66.40 ± 1.83 bc
1.0 mM SA	49.01 ± 5.16 ab	6.67 ± 0.80 a	15.66 ± 1.69 a	17.08 ± 1.86 a	66.57 ± 1.83 bc
2.0 mM SA	44.60 ± 3.99 b	7.56 ± 1.05 a	15.80 ± 1.99 a	21.09 ± 3.25 a	64.44 ± 1.10 c
0.5 mM CA	49.89 ± 3.33 ab	6.49 ± 1.06 a	15.81 ± 1.94 a	17.14 ± 2.08 a	67.90 ± 1.71 ab
1.0 mM CA	51.73 ± 4.66 a	6.53 ± 1.20 a	16.75 ± 1.91 a	17.99 ± 2.14 a	69.50 ± 3.14 a
2.0 mM CA	49.94 ± 3.84 ab	6.77 ± 1.56 a	16.56 ± 2.33 a	17.91 ± 2.70 a	68.02 ± 2.57 ab
0.5 mM MeJA	51.85 ± 5.15 a	6.87 ± 1.30 a	17.42 ± 2.45 a	18.73 ± 2.71 a	68.56 ± 1.71 ab
1.0 mM MeJA	50.04 ± 3.93 ab	6.62 ± 1.03 a	16.37 ± 1.94 a	17.67 ± 2.11 a	67.99 ± 2.00 ab
2.0 mM MeJA	51.64 ± 3.32 a	7.00 ± 1.05 a	17.12 ± 1.80 a	18.50 ± 2.01 a	67.80 ± 1.70 ab
**SP × T**					
Day 7 × Control	51.17 ± 4.78 abc	6.01 ± 0.84 b	14.87 ± 1.61 b	16.04 ± 1.73 b	68.00 ± 1.99 abc
Day 14 × Control	48.96 ± 5.03 abc	6.83 ± 1.19 ab	16.37 ± 2.91 ab	17.74 ± 3.10 ab	67.39 ± 1.57 abc
Day 7 × 0.5 mM PUT	48.67 ± 4.11 abc	6.68 ± 0.60 ab	15.25 ± 1.13 ab	16.66 ± 1.18 ab	66.35 ± 1.70 abc
Day 14 × 0.5 mM PUT	53.11 ± 5.55 ab	7.15 ± 1.35 ab	17.76 ± 3.50 ab	19.15 ± 3.72 ab	67.35 ± 1.66 abc
Day 7 × 1.0 mM PUT	49.86 ± 4.73 abc	6.11 ± 0.66 b	15.62 ± 1.19 ab	16.78 ± 1.28 ab	68.66 ± 1.63 a
Day 14 × 1.0 mM PUT	46.16 ± 3.22 abc	6.67 ± 0.84 ab	15.84 ± 1.99 ab	17.19 ± 2.04 ab	67.14 ± 2.22 abc
Day 7 × 2.0 mM PUT	50.19 ± 2.04 abc	6.00 ± 0.46 b	15.63 ± 0.65 ab	16.75 ± 0.70 ab	68.99 ± 1.30 a
Day 14 × 2.0 mM PUT	50.49 ± 5.92 abc	7.65 ± 1.85 ab	18.03 ± 4.11 ab	19.61 ± 4.44 ab	67.56 ± 1.98 abc
Day 7 × 0.5 mM SA	47.14 ± 2.98 abc	6.94 ± 0.81 ab	16.45 ± 1.37 ab	17.86 ± 1.53 ab	67.16 ± 1.41 abc
Day 14 × 0.5 mM SA	52.26 ± 7.39 ab	8.79 ± 2.34 a	19.22 ± 4.03 a	21.15 ± 4.60 ab	65.64 ± 1.98 abc
Day 7 × 1.0 mM SA	45.61 ± 3.82 bc	6.68 ± 0.52 ab	15.26 ± 1.62 ab	16.64 ± 1.66 ab	66.23 ± 1.37 abc
Day 14 × 1.0 mM SA	52.42 ± 4.00 ab	6.65 ± 1.06 ab	16.07 ± 1.78 ab	17.52 ± 2.07 ab	66.91 ± 2.26 abc
Day 7 × 2.0 mM SA	43.46 ± 3.35 c	7.35 ± 0.51 ab	15.53 ± 1.21 ab	17.18 ± 1.31 ab	64.67 ± 0.43 bc
Day 14 × 2.0 mM SA	45.74 ± 4.49 bc	7.78 ± 1.43 ab	16.06 ± 2.63 ab	24.99 ± 3.51 a	64.20 ± 1.52 c
Day 7 × 0.5 mM CA	48.62 ± 3.02 abc	6.20 ± 0.66 b	15.67 ± 1.49 ab	16.54 ± 1.01 ab	68.22 ± 1.83 ab
Day 14 × 0.5 mM CA	51.16 ± 3.33 abc	6.79 ± 1.35 ab	15.95 ± 2.43 ab	17.74 ± 2.74 ab	67.59 ± 1.67 abc
Day 7 × 1.0 mM CA	52.31 ± 2.24 ab	5.83 ± 0.36 b	15.58 ± 0.78 ab	16.64 ± 0.77 ab	69.51 ± 1.33 a
Day 14 × 1.0 mM CA	51.15 ± 6.42 abc	7.24 ± 1.35 ab	17.92 ± 2.04 ab	19.34 ± 2.26 ab	69.48 ± 4.43 a
Day 7 × 2.0 mM CA	49.70 ± 2.97 abc	6.00 ± 1.34 b	15.19 ± 1.48 ab	16.36 ± 1.82 ab	68.69 ± 3.00 a
Day 14 × 2.0 mM CA	50.18 ± 4.79 abc	7.54 ± 1.44 ab	17.93 ± 2.28 ab	19.46 ± 2.61 ab	67.35 ± 2.06 abc
Day 7 × 0.5 mM MeJA	49.05 ± 2.32 abc	6.16 ± 1.00 b	15.70 ± 1.62 ab	16.88 ± 1.88 ab	68.68 ± 1.48 a
Day 14 × 0.5 mM MeJA	54.64 ± 5.82 a	7.59 ± 1.22 ab	19.13 ± 1.87 a	20.58 ± 2.10 ab	68.44 ± 2.03 ab
Day 7 × 1.0 mM MeJA	47.86 ± 2.66 abc	6.33 ± 0.80 b	15.23 ± 1.31 ab	16.50 ± 1.49 ab	67.50 ± 1.35 abc
Day 14 × 1.0 mM MeJA	52.22 ± 3.92 ab	6.92 ± 1.20 ab	17.50 ± 1.86 ab	18.84 ± 2.06 ab	68.48 ± 2.50 ab
Day 7 × 2.0 mM MeJA	50.73 ± 3.03 abc	6.54 ± 0.84 ab	15.98 ± 1.30 ab	17.26 ± 1.44 ab	67.74 ± 1.87 abc
Day 14 × 2.0 mM MeJA	52.56 ± 3.57 ab	7.46 ± 1.10 ab	18.27 ± 1.51 ab	19.74 ± 1.78 ab	67.87 ± 1.65 abc
**Level of significance**					
SP	**	**	**	**	ns
T	**	ns	ns	ns	**
SP × T	**	**	**	*	**

*Note:* Lowercase letters indicate differences in storage period, treatments and SP × T interaction.

Abbreviations: ±, Standard deviation of mean; CA, Citric acid; MeJA, Methyl jasmonate; ns, non‐significant; PUT, Putrescine; SA, Salicylic acid.

* *p* < 0.05.

** *p* < 0.01.

L* color value expresses the lightness and darkness of the color (higher values indicate lighter color). The L* value on day 14 (50.85) was found to be significantly higher than the L* value on day 7 (48.80). The lightness (L*) of the mushrooms generally tended to increase slightly from day 7 to day 14. This suggests that mushrooms may retain their appearance during the storage period under specific treatments. Among the treatments, 0.5 mM PUT, 1.0 mM CA, 0.5 mM MeJA, and 2.0 mM MeJA were statistically in the same group and had the highest L* values. These treatments significantly increased the L* value compared to the control and may help maintain a lighter color. On the other hand, the lowest L* value was determined in 2.0 mM SA. When the SP × T interaction was examined, the highest L* value (54.64) was determined in 0.5 mM MeJA on the 14th day, while the lowest L* value (43.46) was observed in 2.0 mM SA on the 7th day. CA and MeJA were particularly effective in maintaining or enhancing lightness, suggesting their potential role in mitigating oxidative or enzymatic browning during storage (Table 6).

a* color value represents the red–green coordinate (positive values indicate red; negative values indicate green). a* value increased significantly during storage, indicating a shift toward redder tones. These changes reflect natural aging processes and pigmentation alterations during storage. In the study, the a* color value in *P. ostreatus* mushroom, which was applied to different postharvest treatments and storage periods, varied between 5.83 (Day 7 × 1.0 mM CA)– 8.79 (Day 14 × 0.5 mM SA) (Table 6).

b* color value represents the yellow–blue coordinate (positive values indicate yellow; negative values indicate blue). b* value on day 14 (17.39) was found to be significantly higher than the b* value on day 7 (15.54). It was observed that the b* value increased as the storage period increased. This shows that the color shifted toward a more yellow color over time. When the SP × T interaction was examined, b* value varied between 14.87 (Day 7 × Control) and 19.22 (Day 14 × 0.5 mM SA) (Table 6).

Chroma value indicates color intensity or saturation. Chroma value significantly increased during storage, rising from 16.78 (Day 7) to 19.46 (Day 14). This indicates an increase in color saturation over time. Depending on the postharvest treatments and storage periods considered in the study, chroma values ranged from 16.04 to 24.99. The highest chroma value was determined in 2.0 mM SA on day 14. On the other hand, the control on day 7 was found to have the lowest chroma value (Table 6).

Hue angle represents the actual color tone, calculated from a* and b* values. Hue angle was significantly affected by the treatments. The highest hue angle was determined in 1.0 mM CA (69.50). On the contrary, the lowest hue angle was recorded in 2.0 mM SA (64.44). When the SP × T interaction was examined, hue angle value varied between 64.20 (Day 14 × 2.0 mM SA) and 69.51 (Day 7 × 1.0 mM CA) (Table 6).

Lightness tended to decrease slightly over time for most treatments, indicating potential darkening of the mushrooms during storage. A general trend toward darker and more saturated colors was noted as storage progressed, which could be indicative of the natural degradation of pigments or biochemical changes in the mushrooms. Some treatments (e.g., CA and MeJA) appear more effective in maintaining desirable color properties, and this may be valuable for improving the visual appeal and marketability of mushrooms. Treatments such as CA and MeJA show promise as natural preservatives that can enhance the aesthetic quality of *P. ostreatus*, making them more attractive for commercial applications. The results of this study underscore the importance of optimizing storage conditions and treatment concentrations to preserve the visual appeal of *P. ostreatus*.

Color is one of the most important quality criteria affecting consumer preference in edible mushrooms (Khan et al. 2015). Color changes in mushrooms are mainly related to the oxidative process (Ventura‐Aguilar et al. 2017). Mohapatra et al. (2008) stated that physiological disorders, very high PPO content, and phenolic compounds are effective in the darkening of mushroom color during storage. The effect of compounds such as PUT, SA, CA, and MeJA on postharvest color change in mushrooms is generally related to antioxidant activity, phenolic compound synthesis, enzymatic browning, and cell membrane stability. PUT, SA, and CA generally delay color changes and maintain the L* value. PUT and SA can delay darkening due to their antioxidant effects, prevent pigment loss by protecting the cell membrane, and help preserve color value by reducing PPO activity. Dokhanieh and Aghdam (2016) reported that SA increased antioxidant activity and the accumulation of phenols and was beneficial in reducing postharvest darkening of the mushroom cap by preserving cell membrane integrity. CA can increase color stability by reducing enzymatic browning through its pH regulating effect. Ahvenainen (1996) stated that CA has inhibitory activity on PPO and is effective against browning in processed fruits and vegetables. MeJA can accelerate color changes due to oxidation of phenolic compounds and cause browning by promoting melanin synthesis. Exogenous application of MeJA delays senescence by regulating γ‐aminobutyric acid (GABA) metabolism, which increases stress tolerance and preserves fruit quality, and MeJA leads to a decrease in PPO activity, which helps reduce enzymatic browning (Vaezi et al. 2022).

In the study conducted by Dokhanieh and Aghdam (2016), it was determined that postharvest SA application delayed the darkening of the *A. bisporus* mushroom stored at 4°C for 21 days. Jahangir et al. (2011) determined that postharvest MeJA application in *A. bisporus* mushroom provided better color preservation than control during storage. Meng et al. (2012) stated that postharvest application of MeJA to *A. bisporus* mushroom delayed darkening in mushrooms and increased the L* value compared to the control. In the study conducted by Çavuşoğlu (2018), no significant difference was observed in terms of C* and h° values between the control and MeJA applications at the end of the storage period in *A. bisporus* mushroom stored at 4°C for 20 days, while lower L* values were detected in MeJA applications compared to the control. In the study, it was also determined that L* and h° values decreased significantly compared to the initial values, while the C* value increased. Sarıçam (2008) determined that the color darkening of *A. bisporus* mushroom during storage was significantly less in CA treatment than in the control. In the study conducted by Olotu et al. (2015) on the effects of different doses of CA applications on the postharvest quality characteristics of *P. ostreatus* mushroom, the L* value decreased continuously during the 30‐day storage period at 4°C, while the a* and b* values increased. At the end of the storage period, no significant difference was found between the CA applications and the control in terms of L* and a* values. On the other hand, the b* value was found to be significantly lower in CA applications than in the control. Gupta and Bhat (2016) found that CA treatment significantly reduced the darkening of *A. bisporus* mushroom. In addition, the researchers determined that the L* value decreased continuously during the 12‐day storage period, while the a* and b* values increased. Jia et al. (2018) determined that postharvest PUT application in cucumber affected the color positively and there was no significant difference between control and PUT application in terms of L* and h° color values at the end of the storage period. Kibar et al. (2024) found that the effects of PUT, SA, and CA applications on the L*, b*, and Chroma color values of cauliflower were insignificant. The dosage of compounds such as PUT, SA, CA, and MeJA, duration of application, and storage conditions can significantly affect color change.

### 3.5 Hierarchical Cluster and Principal Component Analysis

**FIGURE 1:**
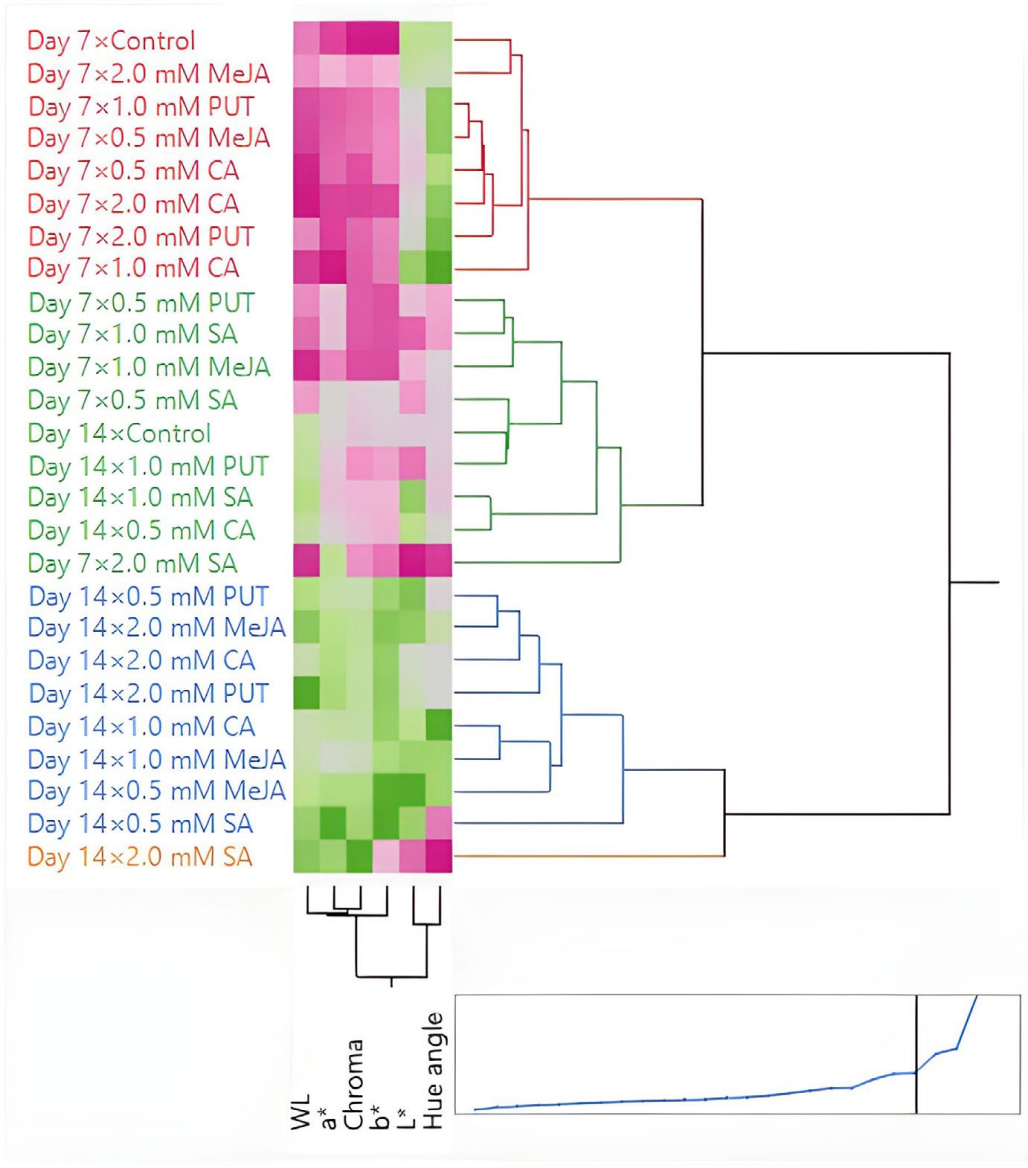
Heatmap visualization results showing the effects of putrescine (PUT), salicylic acid (SA), citric acid (CA), and methyl jasmonate (MeJA) on weight loss (WL), L*, a*, b*, chroma, and hue angle of *P. ostreatus* during storage.

To better understand the effects of exogenous PUT, SA, CA, and MeJA on the postharvest quality parameters of *P. ostreatus* mushrooms, HCA was performed. Control samples (untreated mushrooms) formed a separate and distinct cluster, indicating significant differences in WL and color parameters compared to the treated samples (Figure 1). This highlights the effectiveness of exogenous applications in modulating postharvest changes. PUT and CA treatments (particularly at 1.0 mM and 2.0 mM concentrations) clustered closely, suggesting that these treatments had similar protective effects in reducing WL and preserving color stability. This similarity implies that PUT and CA may share underlying mechanisms in mitigating postharvest deterioration. SA and MeJA treatments formed separate clusters, indicating that they exert different effects on postharvest quality parameters. While SA treatments at higher concentrations (1.0 mM and 2.0 mM) grouped together, MeJA‐treated samples formed more dispersed clusters, suggesting a more variable response in color retention and WL control. Notably, lower concentrations of each treatment (0.5 mM) were often positioned in intermediate clusters, reflecting a moderate influence on postharvest quality compared to higher doses. WL appeared to be a primary distinguishing factor in clustering, with control samples exhibiting the highest WL and clustering separately from treated samples. Color attributes (L*, a*, b*, chroma, hue angle) contributed significantly to treatment differentiation, with PUT and CA treatments clustering closely due to their superior retention of color stability. Chroma and hue angle, which represent color intensity and tonal shifts, respectively, showed strong correlations in clustering, indicating that these attributes were notably affected by specific treatments. The hierarchical clustering results suggest that exogenous applications of PUT, SA, CA, and MeJA significantly influence the postharvest quality of *P. ostreatus*, with distinct effects based on concentration and compound type. The separation of control samples from treated samples demonstrates the efficacy of these bioregulators in delaying deterioration. The clustering patterns also indicate that PUT and CA are more effective in preserving postharvest quality, while SA and MeJA exhibit distinct regulatory effects that may be beneficial under specific conditions.

**FIGURE 2:**
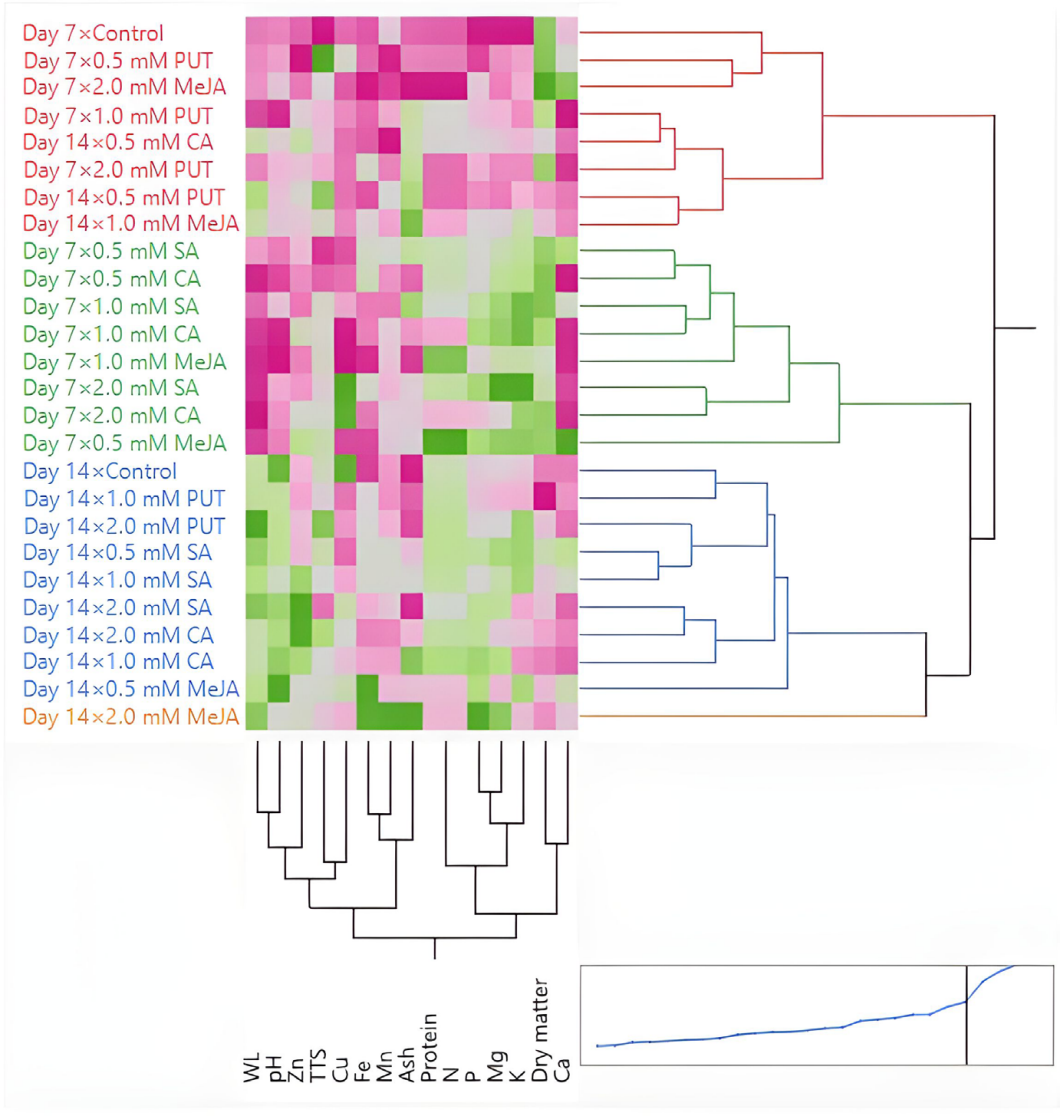
Heatmap visualization results showing the effects of putrescine (PUT), salicylic acid (SA), citric acid (CA), and methyl jasmonate (MeJA) on WL, TTS, proximate, and mineral composition of *P. ostreatus* during storage. Ca, Calcium; Cu, Copper; Fe, Iron; K, Potassium; Mg, Magnesium; Mn, Manganese; N, Nitrogen; P, Phosphorus; TSS, Total soluble solids; WL, Weight loss; Zn, Zinc.

HCA focused on WL, proximate composition, and mineral content, including TSS, N, K, P, Mg, Ca, Fe, Mn, Cu, and Zn. The heatmap visualization in Figure 2 illustrates the clustering of different treatments and storage durations based on these parameters. PUT and CA treatments at 1.0 mM and 2.0 mM concentrations were closely grouped, suggesting their strong effectiveness in preserving proximate and mineral composition while reducing WL. These treatments maintained higher TSS, N, and mineral content, contributing to better postharvest stability. SA and MeJA treatments formed distinct clusters, indicating their differing effects on postharvest parameters. SA‐treated samples at higher concentrations (1.0 and 2.0 mM) tended to group together, implying a concentration‐dependent effect on nutrient retention. Meanwhile, MeJA‐treated samples showed more dispersed clustering, suggesting variable influences on nutrient composition and postharvest stability. Lower concentrations (0.5 mM) of all treatments generally clustered in intermediate groups, signifying partial effectiveness in maintaining postharvest quality, with moderate reductions in WL and nutrient degradation. Proximate composition (nitrogen content) played a significant role in differentiating treatment effects, with PUT and CA treatments maintaining the highest values, leading to their close clustering. Mineral composition (K, P, Mg, Ca, Fe, Mn, Cu, Zn) contributed significantly to cluster separation, highlighting differences in the ability of each treatment to maintain mineral stability. PUT and CA treatments at higher concentrations clustered together, showing the strongest retention of essential minerals, whereas SA and MeJA treatments exhibited more variable effects on mineral content.

**FIGURE 3:**
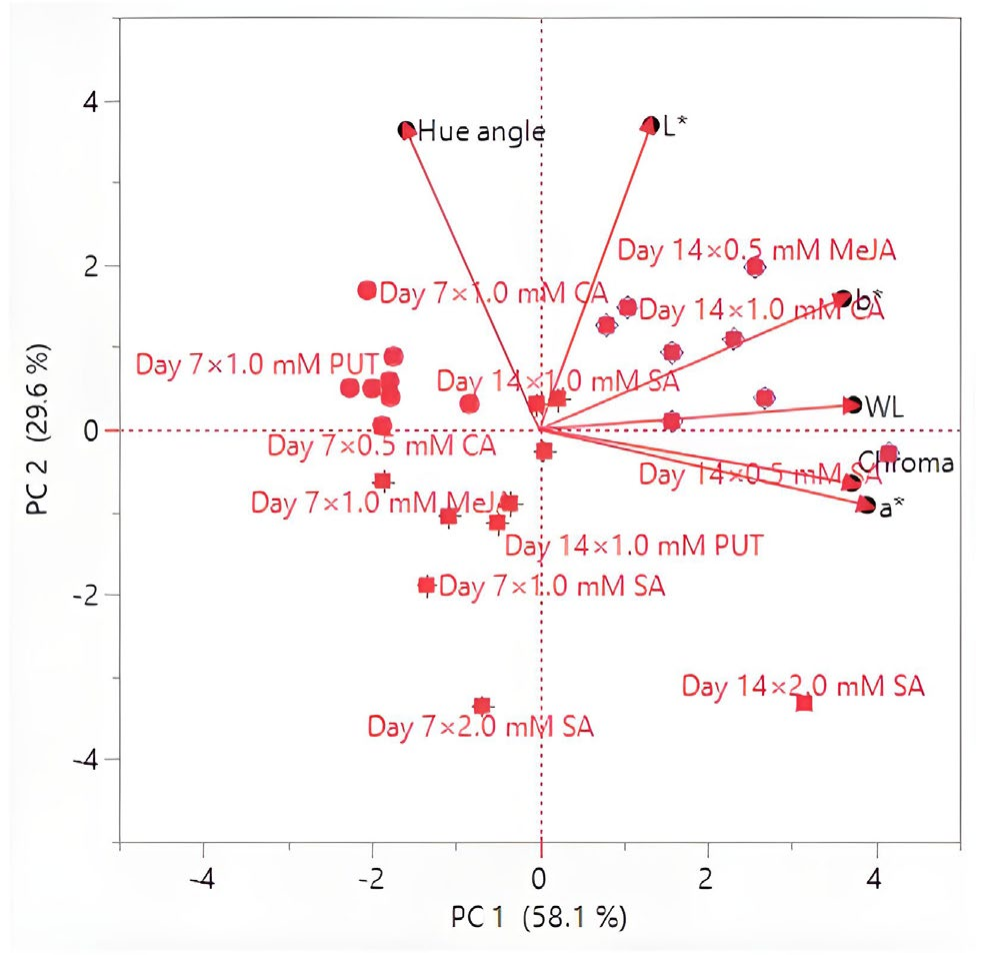
Biplot graph showing the effects of putrescine (PUT), salicylic acid (SA), citric acid (CA), and methyl jasmonate (MeJA) on weight loss (WL), L*, a*, b*, chroma, and hue angle of *P. ostreatus* during storage.

The PCA results indicated that the first two PCs accounted for a substantial proportion of the total variance, with PC 1 explaining 58.1% and PC 2 explaining 29.6%, cumulatively capturing 87.7% of the variability in the dataset (Figure 3). The eigenvalues for PC 1 and PC 2 were 3.4883 and 1.7771, respectively, suggesting that these components effectively summarize the variations observed in the measured parameters. The remaining four components contributed marginally to the overall variance, with PC 3 to PC 6 together accounting for only 12.24% of the total variance. The loading matrix revealed the contributions of different quality attributes to each principal component. PC 1 was primarily associated with WL, a*, b*, and chroma, all showing strong positive loadings. PC 2 was mainly influenced by the huge angle and L*, indicating their substantial role in explaining postharvest quality variations. The biplot representation illustrated the relationships between different quality attributes and storage conditions, confirming that variations in WL, color properties, and chroma were the most significant determinants of postharvest quality across different treatments. On day 7, lower‐dose treatments, particularly 0.5 mM and 1.0 mM SA, CA, and PUT, showed negative coordinates on PC 1, indicating reduced WL and improved color stability compared to the control. Conversely, on day 14, treatments with 2.0 mM PUT and 2.0 mM SA demonstrated a strong positive association with PC 1, reflecting their pronounced effect on maintaining postharvest quality attributes. The untreated control group clustered separately, indicating a distinct degradation pattern over time.

**FIGURE 4:**
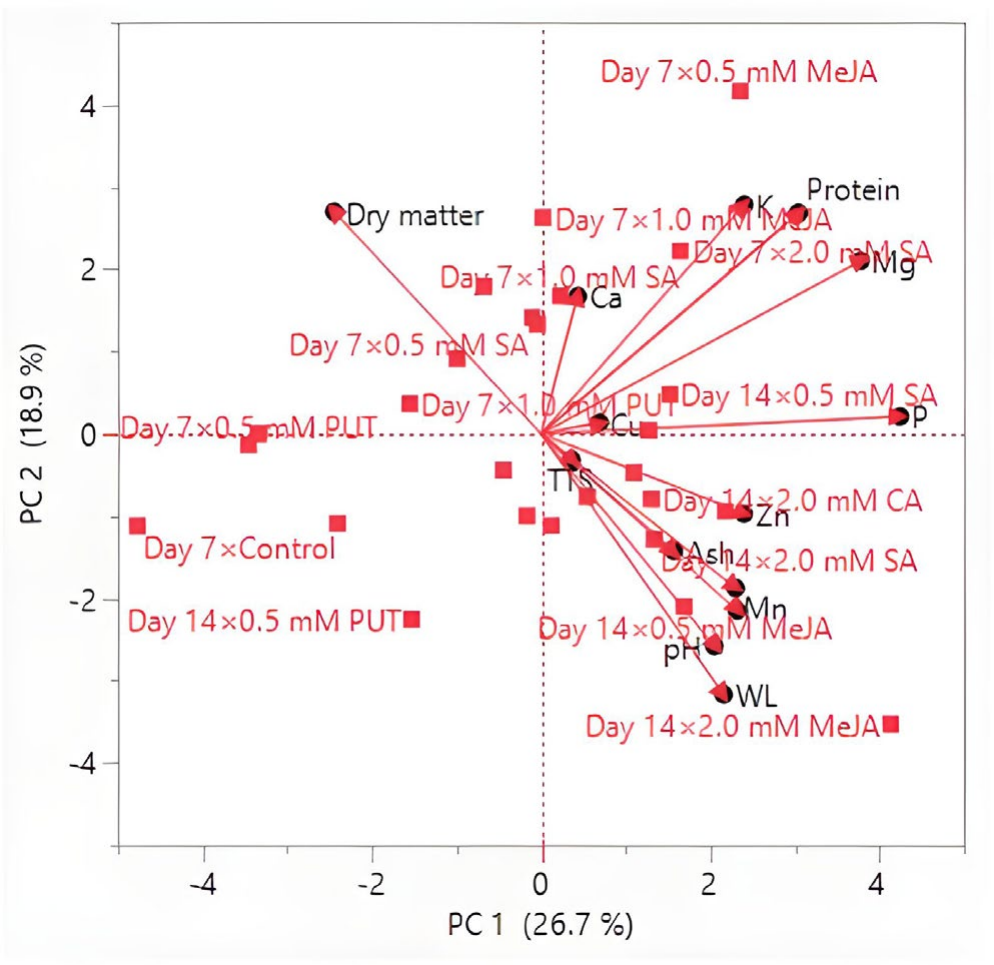
Biplot graph results showing the effects of putrescine (PUT), salicylic acid (SA), citric acid (CA), and methyl jasmonate (MeJA) on WL, TTS, proximate, and mineral composition of *P. ostreatus* during storage. Ca, Calcium; Cu, Copper; Fe, Iron; K, Potassium; Mg, Magnesium; Mn, Manganese; N, Nitrogen; P, Phosphorus; TSS, Total soluble solids; WL, Weight loss; Zn, Zinc.

The PCA results revealed that the first two PCs (PC 1 and PC 2) accounted for a significant portion of the total variation in the dataset (Figure 4). PC 1 explained 26.7% of the variance, while PC 2 explained 18.9%, capturing a cumulative variance of 45.6%. The subsequent components contributed less to the total variance, with PC 3 to PC 6 collectively accounting for an additional 37.58%, leading to a cumulative variance of 83.20%. The remaining components each contributed less than 5% to the overall variability. WL, K, and Fe also contributed substantially to PC 1. PC 2 was primarily influenced by dry matter, Ca, Zn, and pH, reflecting their importance in determining postharvest quality dynamics. The biplot representation demonstrated the relationships among quality attributes and how different treatments influenced these parameters over the storage period. On day 7, low‐dose treatments (0.5 mM) of SA, CA, and MeJA exhibited a higher association with PC 2, indicating their effect on maintaining mineral content and protein stability. However, treatments with 1.0 mM and 2.0 mM PUT were positioned negatively along PC 1, suggesting a reduction in WL and improved quality attributes. Conversely, on day 14, treatments with 2.0 mM MeJA and SA showed a strong positive association with PC 1, indicating their significant role in preserving protein and mineral content under prolonged storage conditions. The untreated control samples were positioned distinctly, signifying greater degradation in postharvest quality attributes compared to the treated groups.

## 4 Conclusion

This study demonstrated that postharvest treatments of PUT, SA, CA, and MeJA significantly influenced the quality characteristics and storage performance of *P. ostreatus* mushroom. Among the treatments, CA and MeJA were the most effective in reducing postharvest quality losses and extending the storage period. These treatments minimized WL, maintained dry matter and protein content, and improved the retention of essential minerals such as K, P, and Zn. Storage duration influenced key quality parameters, with increases in WL, pH, and certain mineral contents over time, while dry matter content declined. The effects of exogenous treatments varied. 0.5 mM MeJA increased protein content by 13.56%, potassium content by 28.19%, and calcium content by approximately threefold compared to control. Additionally, compared with the control, 2.0 mM CA reduced WL by 7.76% and increased zinc content by 57.00%. It has been determined that the postharvest treatments discussed in the study have the potential to be used in extending the storage period and reducing quality losses in the *P. ostreatus* mushroom. In conclusion, 0.5 mM MeJA and 0.5 mM–2.0 mM CA emerged as promising treatments to maintain postharvest mushroom quality. These treatments could be considered as alternative strategies to enhance the shelf life of *P. ostreatus* for commercial applications. The results obtained in this study are expected to contribute to the food industry by minimizing postharvest losses of *P. ostreatus* mushrooms and to provide information for future studies. Considering the economic importance of mushrooms and the difficulties associated with their rapid deterioration, further research is recommended to reveal the mechanisms underlying the effects of these treatments at biochemical and molecular levels.

## Author Contributions

**Bilgehan Şahin:** data curation (equal), investigation (equal), methodology (equal), resources (equal). **Beyhan Kibar:** data curation (equal), funding acquisition (equal), investigation (equal), methodology (equal), project administration (equal), resources (equal), writing – original draft (equal). **Hakan Kibar:** investigation (equal), visualization (equal), writing – original draft (equal).

## Consent

All the authors have approved the article for publication.

## Conflicts of Interest

The authors declare no conflicts of interest.

## Data Availability

Data are available on request from the authors.
